# A Genomic, Evolutionary, and Mechanistic Study of MCR‐5 Action Suggests Functional Unification across the MCR Family of Colistin Resistance

**DOI:** 10.1002/advs.201900034

**Published:** 2019-04-03

**Authors:** Huimin Zhang, Zhiyong Zong, Sheng Lei, Swaminath Srinivas, Jian Sun, Yu Feng, Man Huang, Youjun Feng

**Affiliations:** ^1^ Department of Pathogen Biology & Microbiology and Department of General Intensive Care Unit of the Second Affiliated Hospital Zhejiang University School of Medicine Hangzhou Zhejiang 310058 China; ^2^ Carl R. Woese Institute for Genomic Biology and Department of Biochemistry University of Illinois at Urbana‐Champaign Urbana IL 61801 USA; ^3^ Center of Infectious Diseases West China Hospital Sichuan University Chengdu 610041 China; ^4^ National Risk Assessment Laboratory for Antimicrobial Resistance of Animal Original Bacteria South China Agricultural University Guangzhou 510642 China; ^5^ College of Animal Sciences Zhejiang University Hangzhou Zhejiang 310058 China

**Keywords:** *Aeromonas hydrophila*, colistin resistance, functional unification, lipid A, MCR‐5, phosphatidylethanolamine (PE) cavity, ping‐pong reaction mechanism, transferable resistance

## Abstract

A growing number of mobile colistin resistance (MCR) proteins is threatening the renewed interest of colistin as a “last‐resort” defense against carbapenem‐resistant pathogens. Here, the comparative genomics of a large plasmid harboring *mcr‐5* from *Aeromonas hydrophila* and the structural/functional perspectives of MCR‐5 action are reported. Whole genome sequencing has identified the loss of certain parts of the Tn*3*‐type transposon typically associated with *mcr‐5*, providing a clue toward its mobilization. Phylogeny of MCR‐5 suggests that it is distinct from the MCR‐1/2 sub‐lineage, but might share a common ancestor of MCR‐3/4. Domain‐swapping analysis of MCR‐5 elucidates that its two structural motifs (transmembrane domain and catalytic domain) are incompatible with its counterparts in MCR‐1/2. Like the rest of the MCR family, MCR‐5 exhibits a series of conservative features, including zinc‐dependent active sites, phosphatidylethanolamine‐binding cavity, and the mechanism of enzymatic action. In vitro and in vivo evidence that MCR‐5 catalyzes the addition of phosphoethanolamine to the suggestive 4′‐phosphate of lipid A moieties is integrated, and results in the consequent polymyxin resistance. In addition, MCR‐5 alleviates the colistin‐induced formation of reactive oxygen species in *E. coli*. Taken together, the finding suggests that a growing body of MCR family resistance enzymes are functionally unified.

## 1 Introduction

Antimicrobial resistance (AMR) has appeared as a significant public threat and is triggering a global health crisis.^1^ Worrisomely, a number of previously unidentified antibiotic resistance machineries emerge in multi‐drug‐resistant pathogens. It seems likely that the AMR‐causing human deaths are predicted to reach 10 million per year in 2050s.^2^ Thereby, WHO recommends both an urgent need for a coordinated worldwide response and an improvement in our fundamental understanding of resistance.^3^ Colistin, the cationic antimicrobial cyclic peptide, acts as a final line of defense against severe infections with carbapenem‐resistant *Enterobacteriaceae*.^4^ However, the renewed interest of colistin in clinical sector seems to be challenged greatly by the global discovery of transferable colistin resistance determinant MCR‐1.^5^ Unlike rare cases of natural/intrinsic colistin resistance which are frequently associated with mutations in chromosomal genes like *phoP‐phoQ* two‐component system^6^ and the regulator gene *mgrB*,^7^ the prevalent plasmid‐borne MCR‐1 mechanism mainly relies on its enzymatic ability to decorate the lipid A moieties of bacterial outer membrane lipopolysaccharides (LPSs), the initial target of colistin.^8^, ^9^ Furthermore, Xu et al. established a working model of a “ping‐pong” reaction exploited by MCR‐1/2^10^, ^11^ and its Neisseria paralog EptA.^11^

Since its first discovery in southern China, in late 2015,^12^ *mcr‐1* has been detected across over 50 countries covering six of seven continents.^13^ A number of diversified bacteria have been found to disseminate *mcr‐1*, most of which are *E. coli* and *Klebsiella pneumoniae*.^13^, ^14^ In addition to the unusual cases located on the bacterial chromosome, *mcr‐1* is predominantly carried on plasmids with diversified replicons (such as IncI2,^15^, ^16^ IncX4,^9^, ^16^, ^17^ and even hybrid versions IncX3‐X4^18^ and IncI2‐IncFIB^16^). During the formulation of this paper, a growing body of new members [namely, MCR‐1,^9^, ^12^, ^19^, ^20^ MCR‐2,^21^, ^22^ MCR‐3,^23^, ^24^ MCR‐4,^25^, ^26^, ^27^ MCR‐5,^25^, ^26^, ^28^ MCR‐6 (renamed from the former *mcr‐2.2* variant^29^), MCR‐7,^30^ and MCR‐8^31^] have been assigned to the MCR family. This indicates an unexpected diversity and ongoing evolution of MCR determinants under some unknown selection. Unlike MCR‐1^13^, ^14^ and MCR‐3,^23^, ^32^, ^33^, ^34^ two prevalent members featuring a dozen heterogeneous variants, the diversity of *mcr‐2* variants might be underestimated, due to the limited availability of its epidemiological investigations.^35^ Since its initial identification in Germany,^28^ *mcr‐5* has been extended to several other countries including Japan,^36^ Spain,^37^ and China.^38^ In general, the *mcr‐5* carriers are ColE1(ColE2)‐type plasmids with a broad host range. Apart from *E. coli* and *Salmonella enterica*, two additional bacterial reservoirs were detected for *mcr‐5*, namely, *Pseudomonas* and *Aeromonas hydrophila*.^38^ Given the fact that (i) certain species of *Aeromonas*, the pathogens of aqua‐cultured fishes have been found to harbor the prevalent *mcr‐3* 39 and (ii) the indiscriminate and unregulated use of colistin in aquaculture production, we believe that the recent discovery of *mcr‐5* in *A. hydrophila* raises crucial questions about its evolutionary origins.

Though MCR‐5 shares low similarity with the paradigmatic MCR‐1 of the MCR family (Figures S1A,B, Supporting Information), it is still thought to function as a PEA‐lipid A transferase. However, little or no information is available about the functional, structural, and mechanistic aspects of MCR‐5. In this study, we aim to provide a composite picture of MCR‐5 from this perspective while also tracing its evolutionary relationships with the other members of the MCR family. The finding might allow us to potentially develop better strategies to manage its spread and to eventually develop therapeutic agents that can reverse colistin resistance.

## 2 Results

### 2.1 Discovery of a New *mcr‐5*‐Harboring Plasmid from *A. hydrophila*

**Figure 1:**
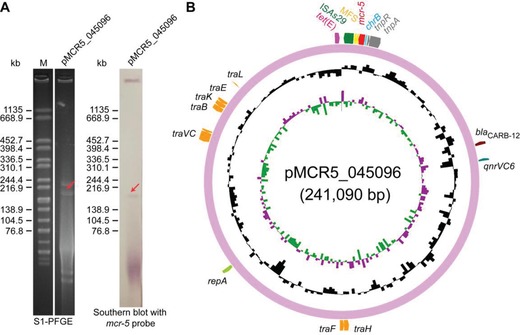
A new *mcr‐5*‐harboring plasmid from *Aeromonas hydrophila*. A) Use of Southern blot to estimate the size of the newly identified *mcr‐5*‐harboring plasmid pMCR‐5_045096 following the separation with SI‐PFGE. B) Circular illustration for genomic map of pMCR‐5_045096. *mcr‐5* is indicated in red.

**Figure 2:**
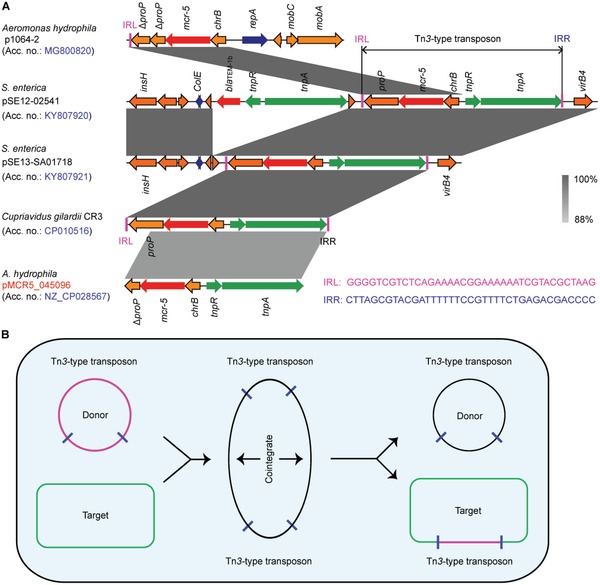
Genomic context of *mcr‐5*‐containing plasmids and genetic analyses for *mcr‐5* dissemination. A) Colinear analyses for genetic environment of *mcr‐5*‐neighboring loci from different plasmid reservoirs or chromosome. Linear comparison of the *mcr*‐*5*‐carrying plasmids p1064‐2 (MG800820), pSE12‐02541 (KY807920), pSE13‐SA01718 (KY807921), chromosome fragment of *C. gilardii* strain CR3, and plasmid pMCR5_045096 (CP028567) was performed in this study. Boxed arrows represent the position and transcriptional direction of ORFs. Regions of >99% identity are marked by gray shading. Genes associated with replication associated genes are colored dark blue, antibiotic resistance genes are colored red, insertion sequences are colored green, and other genes are colored orange. IRL, terminal inverted repeats of left. IRR, terminal inverted repeats of right. B) Scheme for the replicative transposition cycle of Tn*3*‐type transposons harboring *mcr‐5*. The black circle represents the donor ColE‐like plasmids carrying *mcr‐5*‐Tn*3*‐type transposons. The rectangle represents the target of Tn*3*‐type transposons.

A novel 241 kb plasmid, pMCR5_045096, carrying *mcr‐5* was identified by the whole genome sequence of strain WCHAH045096 (**Figure** **1**A,B), which is resistant to >512 µg mL^−1^ of colistin. Though this strain was subsequently identified as *A. hydrophila*, no known plasmid replicon type (Figure 1B) could be associated with pMCR5_045096. Despite repeated attempts of conjugation experiments, mating failed to produce trans‐conjugants on agar plates with colistin and azide, suggesting that this *mcr‐5*‐bearing plasmid pMCR5_045096 is not self‐transmissible. *mcr‐5* and its genomic context are highly similar to that in plasmids pSE12‐02541 and pSE13‐SA01718, where *mcr‐5* was originally identified.^28^ All three plasmids contain a Tn*3*‐like transposon (**Figure** 1B and **2**),^40^ which mediates transposition by a “copy‐in” or “paste‐and‐copy” mechanism^41^ and cointegrates with multiple copies of the transposon sandwiching the target sequence (Figure 2B). However, there are several differences between the transposons in pMCR5_045096 and pSE12‐02541/pSE13‐SA01718 (Figure 2A). First, unlike pSE12‐02541 and pSE13‐SA01718, pMCR5_045096 lacks a characteristic 5 bp direct repeat (DR; ATGTA) that flanks the transposon (Figure 2A). Second, while the transposase (*tnpA*) and the resolvase (*tnpR*) genes share only 86 and 81% nucleotide identity, respectively, between the two transposons (Figure 2A), a nearly identical sequence is observed extending from the left inverted repeat (IRL) to the resolvase binding site I (*res*I). This suggests that a resolvase‐mediated site‐specific recombination has occurred (Figure 2B). Third, an insertion sequence, IS*As29*, was found in one inverted repeat (IR) on pMCR5_045096 flanked by a 5 bp DR (AGACG). In fact, the Tn*3*‐type transposon from pMCR5_045096 is 99% identical to that on the chromosome of *Cupriavidus gilardii* strain CR3, with *mcr‐5* disrupting *proP* without IRL or IRR (Figure 2A). A similar truncated *proP* gene with a half‐formed IR was observed in a porcine *A. hydrophila* isolate from China,^38^ suggesting a multivariate evolution of the plasmids harboring *mcr‐5*. The detail of its evolutionary route, however, remains elusive.

### 2.2 Phylogeny of MCR‐5

**Figure 3:**
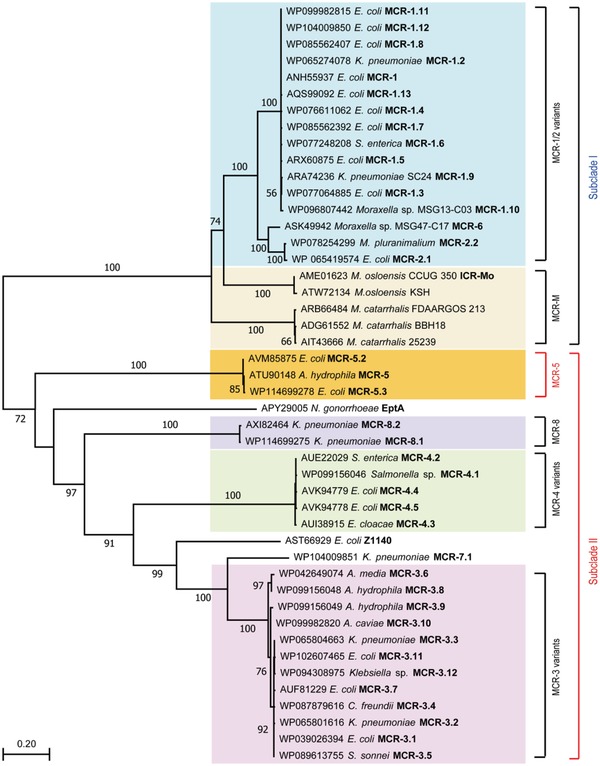
Phylogeny of MCR‐5. An unrooted phylogenetic tree of MCR‐5 and its close homologs is presented with two distinct subclades (Subclade I and Subclade II) with paraphyletic branches. Full protein sequences of MCR enzymes were applied to generate phylogenic tree. Subclade I contains MCR‐1/2 variants (light pink) and its progenitors MCR‐M (light orange), whereas Subclade II comprises MCR‐5 (in yellow), MCR‐4 variants (light blue), and MCR‐3 variants (in green). MCR‐5 is highlighted in bold red font. Abbreviations: *E. coli*, *Escherichia coli*; *K. pneumoniae*, *Klebsiella pneumoniae*; *S. enterica*, *Salmonella enterica*; *M. pluranimalium*, *Moraxella pluranimalium*; *M. osloensis*, *Moraxella osloensis*; *M. catarrhalis*, *Moraxella catarrhalis*; *N. gonorrhoeae*, *Neisseria gonorrhoeae*; *A*. *media*, *Aeromonas media*; *A. hydrophila*, *Aeromonas hydrophila*; *A*. *caviae*, *Aeromonas caviae*; *C. freundii*, *Citrobacer freundii*; *S. sonnei*, *Shigella sonnei*.

The molecular phylogeny of MCR‐5 and other members of the MCR family, constructed by the maximum likelihood method, suggests a broad partitioning into two distinct clades (**Figure** **3**). MCR‐5.1 and its variant MCR‐5.2 share a common ancestor with the larger MCR‐3 and MCR‐4 family. This is further supported by the fact that both MCR‐5.1 and a large number of MCR‐3 variants have been observed in *Aeromonas* species. Together they form a phylogenetic group that is distinct from the tightly clustered MCR‐1 and MCR‐2 families. MCR‐1/2 are also clustered with the ICR‐Mo family from *Moraxella* which is thought to represent a chromosomal reservoir of genetic diversity for the MCR‐1/2 family. Interestingly, the intrinsic colistin resistance determinant EptA, from the naturally colistin resistant *Neisseria* sp. is more closely related to MCR‐3/4 than it is to MCR‐5. A better understanding of these evolutionary relationships requires an extensive genetic and functional characterization of members of the MCR family.

### 2.3 Characterization of MCR‐5 and Its Action

**Figure 4:**
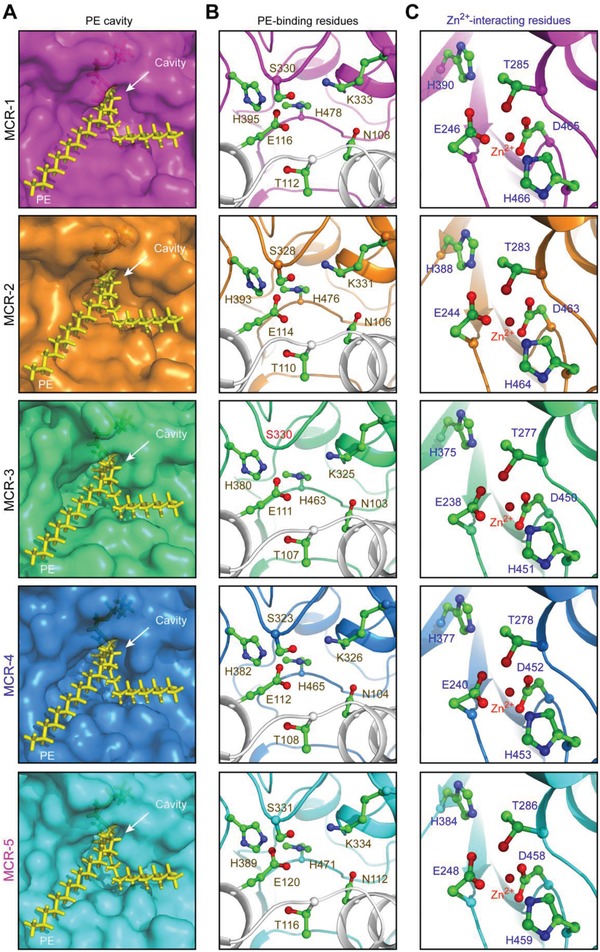
Parallels among PE‐recognizable cavities of the MCR family of lipid A modifiers. A) Similarity of PE cavities in different members of MCR family. B) Visualization for a conserved motif comprising 7 PE‐binding residues. C) Parallels in zinc‐binding residues of MCR‐5 and MCR‐1/2/3/4. PyMol is applied to generate the photographs of surface structure, PE cavities, and enzymatically catalytic center.

MCR‐5 was modeled using EptA as a template and is predicted to be an integral membrane protein (Figure S1, Supporting Information) with two distinct domains connected by a flexible linker, a periplasmic catalytic domain, and a transmembrane domain comprised of five α‐helices (Figure S2A,B, Supporting Information). The N‐terminal hexa‐histidine‐tagged MCR‐5 protein in full length was purified to homogeneity (Figure S3A, Supporting Information) with an apparent molecular mass of 63.34 kDa (Figure S3B, Supporting Information) and verified by peptide‐mass fingerprinting with a coverage of 91.04% (Figure S3C, Supporting Information). MCR‐5 behaves as a monomer in solution when examined using size exclusion chromatography (Figure S4, Supporting Information). A circular dichroism spectrum of the purified protein shows a peak followed by a dip characteristic of an alpha helix rich protein (Figure S3D, Supporting Information). The presence of bound zinc was confirmed by inductively coupled plasma‐mass spectrometry (ICP‐MS, Figure S3E, Supporting Information). Further, the interaction of the physiological substrate lipid‐PE with MCR‐5 was predicted by molecular docking using the modeled structure of MCR‐5 (Figure S2C,D, Supporting Information). This reveals a cavity at the interface of the two domains that perfectly accommodates the PE head group of the substrate (**Figure** **4**A). In fact, the head group is also observed to interact with a bound zinc at the end of the active site cavity (Figure 4B,C). This suggests a common structural architecture across the MCR family (at least from MCR‐1 to MCR‐5, Figure 4).

**Figure 5:**
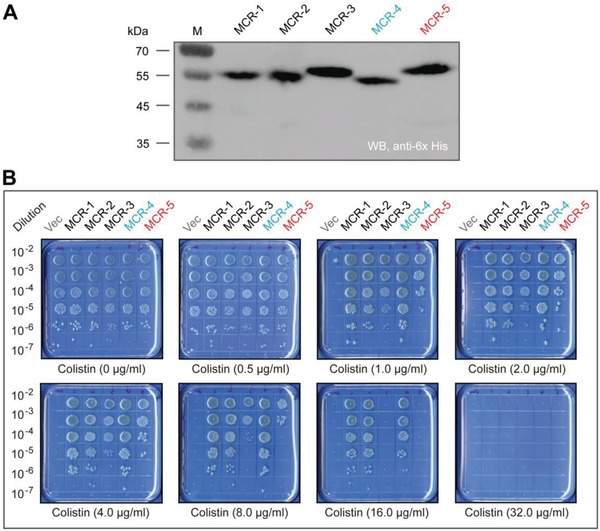
Comparative analyses for colistin resistance levels in *E. coli* conferred by an array of different MCR versions. A) Western blot‐aided comparative analyses of functional expression of *mcr‐1/2/3/4/5* in vivo. B) Growth viability of *E. coli* harboring different version of MCR family of enzymes on the LBA plates supplied with varied level of colistin. A representative result is given from three independent experiments. Designation: Vec, pBAD24; WB, Western blot.

**Figure 6:**
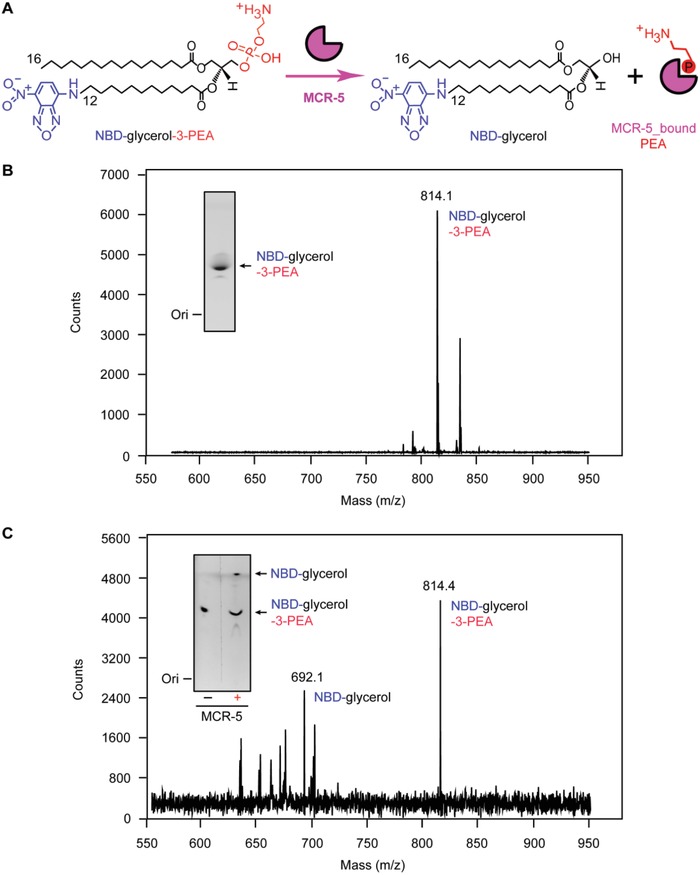
Enzymatic action of MCR‐5 in vitro. A) Scheme for chemical reaction of MCR‐5 in hydrolyzing an alternative lipid substrate of PE, NBD‐glycerol‐3‐PEA, into NBD‐glycerol and an adduct of MCR‐5_bound PEA. B) LC/MS identity of the alternative lipid substrate of PE, NBD‐glycerol‐3‐PEA. C) LC/MS‐based detection for the mixture of MCR‐5 reaction with NBD‐glycerol‐3‐PEA as substrate. Inside gel separately refers to TLC assays for the substrate of NBD‐glycerol‐3‐PEA (B) and its resultant product NBD‐glycerol (C).

Western blot illustrated that all five MCR proteins express well in a susceptible *E. coli* host (**Figure** **5**A). When expressed as a hexa‐histidine tagged protein from a plasmid, MCR‐5 confers resistance to 8 µg mL^−1^ of colistin, which is comparable to that of MCR‐3 (Figure 5B). In comparison, strains expressing MCR‐1, MCR‐2, or MCR‐4 are resistant to around 16 µg mL^−1^ of colistin (Figure 5B). This suggests that despite their structural unification (Figure 4), the divergence of MCR‐like enzymes exists in the context of antibiotic resistance (Figure 5). To test the activity of MCR‐5 in vitro, a fluorescently labeled substrate, NBD‐glycerol‐3‐PEA was incubated with purified MCR‐5 enzyme. MCR‐5 is observed to cleave off the PE group substitute, NBD‐glycerol‐3‐PEA and release NBD‐glycerol (**Figure** **6**A,B) when separated on a thin layer chromatography (TLC) plate from the reactant (Figure 6C). Both reactants and products were verified by mass spectrometry (Figure 6B,C).

### 2.4 Functional Dissection of MCR‐5 Colistin Resistance

**Figure 7:**
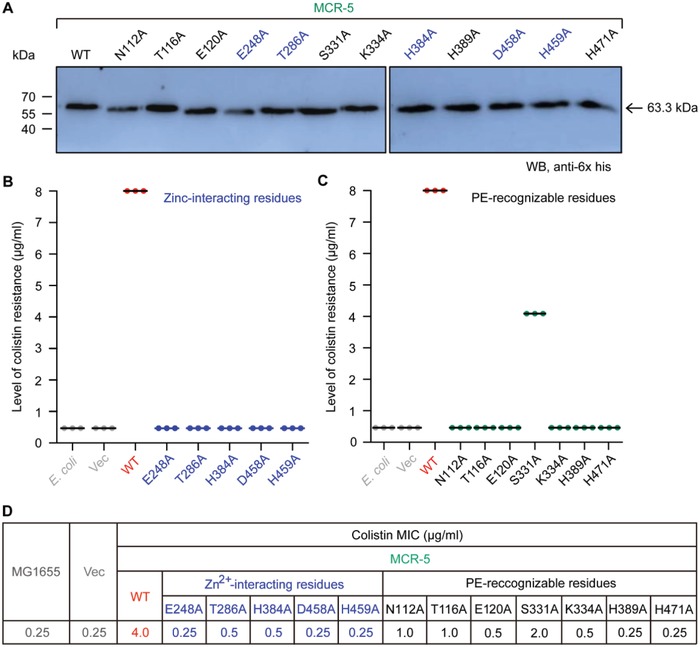
Mapping genetic elements necessary for MCR‐5 colistin resistance. A) Western blot‐based expression assays for MCR‐5 and its 12‐point mutants in *E. coli*. B) Site‐directed mutagenesis analyses for the Zn^2+^‐binding motif of MCR‐5 in the context of colistin resistance using the colistin susceptibility tests. The five residues in Zn^2+^‐binding motif of MCR‐5 denote E248, T286, H384, D458, and H459, respectively. C) Colistin susceptibility‐based dissection of the PE‐interactive residues of MCR‐5. The seven residues denote N112, T116, E120, S331, K334, H389, and H471, respectively. Assays of three individual bacterial viability on colistin agar plates were conducted. D) Minimum inhibitory concentration (MIC) of colistin of *E. coli* harboring *mcr‐5* and/or its point mutants. Designation: Vec, pBAD24; WT, wild‐type.

The active site cavity and the adjacent zinc binding site of MCR‐5 were elucidated by docking the physiological substrate to the modeled structure. In total, 12 residues were identified (Figure 4), which consist of seven residues (N112, T116, E120, S331, K334, H389, and H471, in Figure 4C) that interact with PE substrate, and five residues that might interact with zinc (H384, T286, E248, D458, and D459, in Figure 4B). This suggests that both the shape and the composition of the active site cavity is conserved across the entire MCR family (Figure 4). To test the essentiality of these residues, alanine mutants of these 12 residues were generated in MCR‐5 and then tested in vivo for their ability to confer colistin resistance to *E. coli*. Though all mutants expressed as verified by Western blotting (**Figure** **7**A), 11 of the 12 mutants were inactive in bacterial viability assays on the colistin LBA plates (Figure 7B,C), with only S331A retaining partial activity (≈50%, Figure 7C). More subtle differences between the residues were observed during minimum inhibitory concentration (MIC) measurements with S331 being resistant to colistin (2 µg mL^−1^), N112A and T116A having significantly reduced susceptibility to colistin (1 µg mL^−1^), T286A, H384A, E120A, and K334A having reduced susceptibility to colistin (0.5 µg mL^−1^) and E248A, D458A, H459A, H389A, and H471A being as susceptible as the negative control (0.25 µg mL^−1^) (Figure 7D).

To further examine the functionality of MCR‐5 and its mutants, their ability to modify the lipid A of a susceptible host *E. coli* MG1655 strains was tested (Figure S5, Supporting Information). Purified lipid A extracts were subjected to mass spectrometry. The strain expressing MCR‐5 has a peak (*m*/*z*, 1919.378, in Figure S5C, Supporting Information) that is 123u more than that found in the wild‐type *E. coli* MG1655 (*m*/*z*, 1796.274–1796.743, in Figure S5A,B, Supporting Information). This corresponds to an addition of a PEA group to lipid A resulting in PPEA‐4′‐Lipid A. In agreement with the colistin MIC trials (Figure 7D), the mutant S331A could successfully modify lipid A (*m*/*z*, 1920.198) when evaluated by mass spectrometry (Figure S5I, Supporting Information). A similar result is also observed for N112A and T116A (Figure S5D,E, Supporting Information). All other mutants could no longer modify lipid A in vivo and had wild‐type lipid A species (Figure S5F–H and J–O, Supporting Information). Together, the data suggest that the residues S331A, N112A, and T116A (Figure S5D,E,I, Supporting Information) might not be directly involved in the catalytic mechanism illustrated with a “ping‐pong” reaction model (Figure S6, Supporting Information) and are at least partially dispensable to function. These residues might instead be involved in stabilizing the substrate.

### 2.5 Interdomain Interaction of MCR‐5

**Figure 8:**
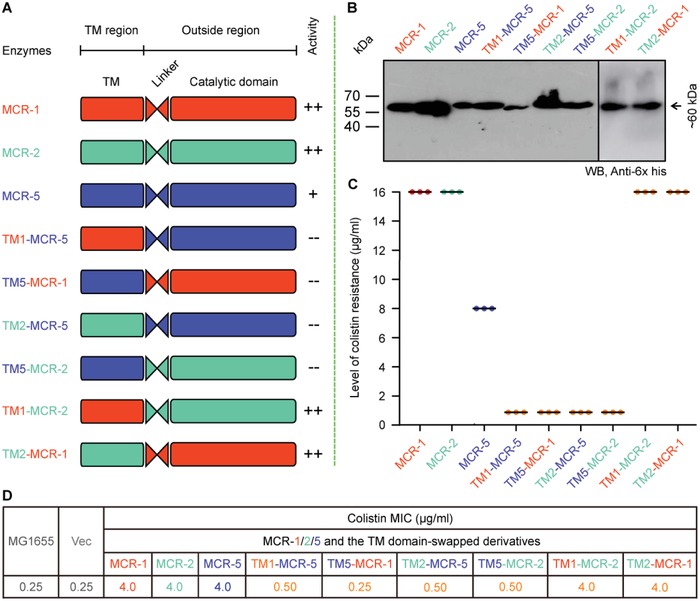
Domain‐swapping analyses of MCR‐1, MCR‐2, and MCR‐5. A) Scheme for domain‐swapped constructs between MCR‐5 and MCR‐1/2. B) Western blot‐based confirmation for functional expression of *mcr‐5* and its hybrid versions in *E. coli*. C) Bacterial viability of *E. coli* expressing *mcr‐5* and its hybrid derivatives on the LBA plates supplied with colistin. Three independent tests were performed. D) Colistin MIC of *E. coli* MG1655 harboring the wild‐type of *mcr‐5* or its hybrid derivatives. In total, six derivatives from domain‐swapping among MCR‐1, MCR‐2, and MCR‐5. Designations: Vec, pBAD24; TM1‐MCR‐5, a derivative of MCR‐5 with TM1 region of MCR‐1 in place of its native TM domain; TM5‐MCR‐1, a hybrid version of MCR‐1 whose TM region is replaced with the counterpart in MCR‐5; TM2‐MCR‐5, a mosaic version of MCR‐5 whose TM region is exchanged with that of MCR‐2; TM5‐MCR‐2, a hybrid derivative of MCR‐2 whose TM region is replaced with that of MCR‐5; TM1‐MCR‐2, a hybrid derivative of MCR‐2 whose TM region is replaced with that of MCR‐1; and TM2‐MCR‐1, a derivative of MCR‐1 whose TM region is replaced with that of MCR‐2.

Like the other MCR enzymes, MCR‐5 has two domains connected by a flexible linker (**Figure** **8**A). Given that MCR‐1 and MCR‐5 are evolutionarily distinct (Figure 3), the intercompatibility of their domains was compared to that between the more closely related MCR‐1 and MCR‐2 (Figure 8A). As expected, the transmembrane and catalytic domains of MCR‐1 and MCR‐2 are perfectly interchangeable (Figure 8A–D). Both chimeric proteins (TM1‐MCR‐2 and TM2‐MCR‐1) are active in both bacterial viability assays on colistin LBA plates (Figure 8C) and colistin MIC measurements (Figure 8D) and can modify lipid A when examined by mass spectrometry (Figure S7J,K, Supporting Information). However, the domains of MCR‐1/2 and MCR‐5 are mostly incompatible (Figure S7A–I, Supporting Information). Western blot result argues that this incompatibility between MCR‐5 and MCR‐1/2 is due to insufficient expression of the chimeric *mcr* derivative (Figure 8B). This is generally consistent with no detectable activity (0.25–0.5 µg mL^−1^) in the functional assay of colistin resistance with TM1‐MCR‐5, TM2‐MCR‐5, and TM5‐MCR‐2 (Figure 8C,D). Despite the overall similarity in the architecture and active site on the enzymes (Figure 4), MCR‐5 and MCR‐1/2 have different interdomain relationships that maintain their catalytic activities (Figure 8).

### 2.6 Physiological Role of MCR‐5

**Figure 9:**
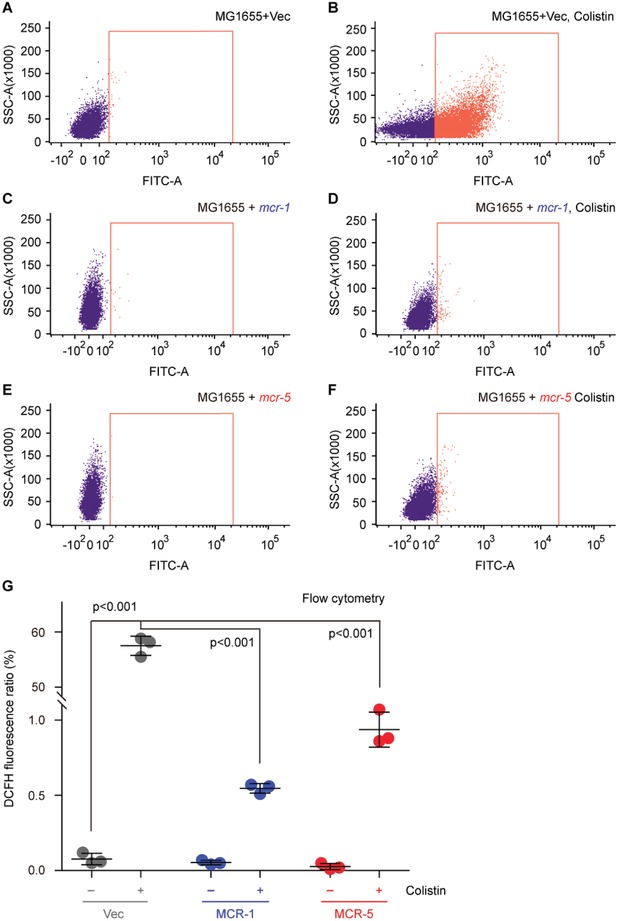
FACS analyses of colistin‐induced ROS level in *E. coli*. A,B) The colistin treatment boosts the accumulation of ROS in *E. coli* with empty vector. C,D) The presence of colistin cannot promote efficient formation of ROS in *mcr‐1*‐harboring in *E. coli*. E,F) The expression of MCR‐5 catalyzes the attachment of PEA to the suggestive 4′‐phosphate position of lipid A anchored on *E. coli* surface and prevents efficient production of intracellular ROS. G) Use of flow cytometry to measure the relative level of ROS in *E. coli* alone or carrying *mcr‐1*/*5*. Flow cytometry of ROS was performed with a BD FACSVerse flow cytometer in which around 10 000 cells are counted at a flow rate of 35 mL min^−1^. The fluorescence of the dye DCFH2‐DA was excited with a 488 nm argon laser and emission was detected with the FL1 emission filter at 525 nm using FL1 photomultiplier tub. The minus symbol denotes the absence of colistin, and the plus symbol refers to the addition of colistin. The data were expressed using one‐way analysis of variance (ANOVA) followed by Tukey–Kramer multiple comparisons post hoc test.^47^ Statistical significance was set at *p* < 0.001. Designations: Vec, pBAD24; “−,” no addition of colistin; “+,” addition of colistin.

**Figure 10:**
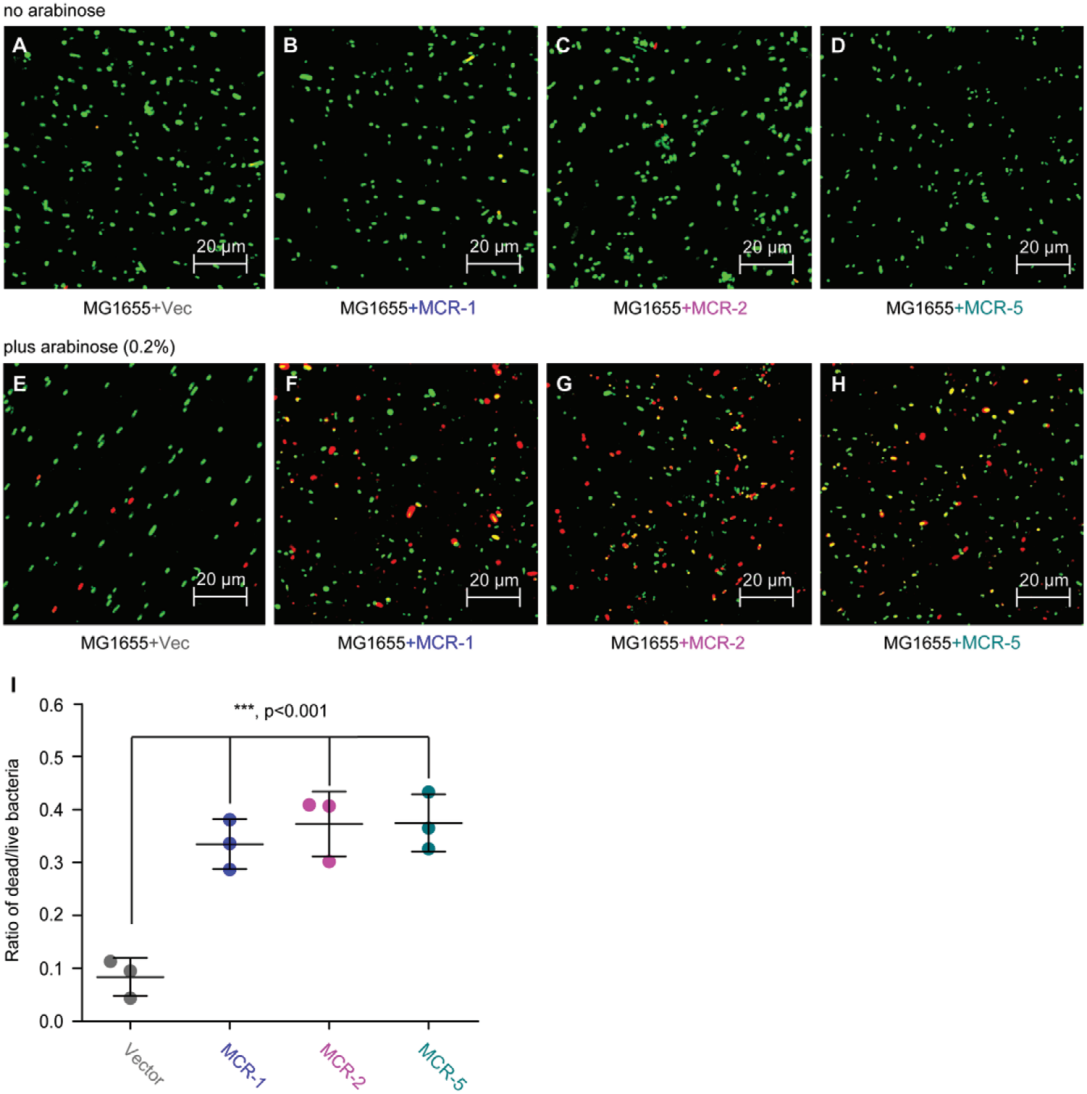
Functional expression of *mcr‐1/2/5* genes is accompanied with bacterial metabolic fitness. Regardless of the presence of the A,E) empty vector pBAD24 or B–D) MCR‐1/2/5, no addition of the inducer arabinose cannot significantly alter bacterial survival in *E. coli* MG1655. F–H) Confocal microscopy assays illustrate that arabinose (0.2%)‐triggered expression of MCR‐1/2/5 interferes bacterial viability. I) Measurement of the relative ratio of LIVE/DEAD *E. coli* strains expressing MCR‐1/2/5. 0.2% (w/v) l‐arabinose was added to initiate the expression of *mcr‐1/2/5*. Bacterial cells were stained with LIVE/DEAD kit, giving the images with confocal laser scanning microscopy. Green and red refer to live and dead cell. Vector refers to pET21. The data were expressed using one‐way analysis of variance (ANOVA) followed by Tukey–Kramer multiple comparisons post hoc test.^47^ Statistical significance was set at ***p < 0.001.

It has been shown that bacterial killing by the antimicrobial peptide colistin involves the hydroxyl radical death pathway activated by antibiotic stimulation.^42^, ^43^ Despite that MCR‐1/2 [plus its progenitor ICR‐Mo (also designated as MCR‐M)]^44^ and the distinct member MCR‐3^23^ have been found to efficiently interfere ROS production in *E. coli* stressed with colistin, it remains unclear as for the in vivo performance of the newly identified member MCR‐5. To address this question, we performed fluorescence activated cell sorting (FACS) analyses (**Figure** **9**) as well as confocal microscopy (Figure S8A–G, Supporting Information). FACS analyses show that the production of colistin‐triggered ROS in the MCR‐5‐expressing *E. coli* (Figure 9E–G) is tens of folds less than that of the control strain MG1655 alone or carrying the empty vector (Figure 9A,B). This is generally consistent with the scenarios in *E. coli* carrying *mcr‐1* (Figure 9C,D), as well as those of MCR‐3,^23^ MCR‐4,^45^ and MCR‐1/2 plus its progenitor ICR‐Mo^44^ observed with confocal microscopy. Confocal microscopy visualization of bacterial viability (LIVE/DEATH) shows that (i) despite carrying pBAD‐borne *mcr*‐*1/2/5* genes, bacterial survival of different *E. coli* strains is pretty good in the normal (uninduced) condition (**Figure** **10**A–E); (ii) the addition of 0.2% arabinose into media efficiently induces the expression of *mcr*‐like genes [namely, *mcr‐1* (Figure 10F), *mcr‐2* (Figure 10G), and *mcr‐5* (Figure 10H)], and promotes/triggers bacterial metabolic stress‐associated death (Figure 10F–H); and (iii) as the direct consequence of MCR‐1/2/5 expression, bacterial MCR‐metabolic fitness (ratio of DEATH/LIVE) is calculated to be 35–40% (Figure 10I).

To further verify this question, we also applied chemical rescue trials (Figure S9, Supporting Information). Obviously, no growth of *mcr*‐negative *E. coli* appears on LBA plates stressed with colistin (Figure S9A–C, Supporting Information), whereas the presence of MCR‐5 confers the ability of the recipient strain to resist bacterial killing by colistin (Figure S9B,C, Supporting Information). Intriguingly, in the presence of bipyridine, a chelator of ferric involved in Fenton reaction, the colistin‐susceptible *E. coli* MG1655 can be efficiently restored from colistin‐mediated cell death (Figure S9C, Supporting Information). In addition, the ROS scavenger, l‐cysteine alone (or together with bipyridine) also significantly rescue bacterial survival of *E. coli* stressed with colistin (Figure S9C, Supporting Information). Evidently, not only does the Fenton reaction participate in the formation of free hydroxyl radicals (Figure S9A,B, Supporting Information) but also functional expression of *mcr‐5*, an evolutionarily distinct genetic determinant of MCR family, quenches/interferes/prevents/terminates the entry of the recipient *E. coli* into the hydroxy radical death pathway (Figure S9B,C, Supporting Information). In general agreement with the statement of *mcr‐1* by Yang et al.,^46^ our results also elucidated that the retardation of bacterial growth is not in the MG1655 strain alone (Figure S10A,B, Supporting Information), but correlated with the reduced survival ratio to adapt the expression of *mcr‐1/2* (Figure S10C,D, Supporting Information) and *mcr‐5* (Figure S10E, Supporting Information). In conclusion, we demonstrated that MCR‐5 modifies lipid A, an important component of bacterial surface structure, stops the penetration of polymyxin into cells, alleviates the formation of ROS, and in turn bypasses (in part, if not all) antibiotic killing by colistin (Figure S8, Supporting Information). This process in MCR‐harboring *E. coli* has an appreciable fitness cost, that is, linking of retarded growth caused by MCR “poisoning” (Figure S10, Supporting Information).

## 3 Discussion

The growing and evolving family of colistin resistance genes is a significant threat to global health. The identification of MCR‐3, MCR‐4, and now MCR‐5 has suggested an evolutionary path that is different from the prevalent MCR‐1/2 family (Figure 3). Though MCR‐3, MCR‐4, and a large number of their variants have been found worldwide, MCR‐5 has only been identified in a few countries in addition to Germany, where it was originally found and only in *E. coli*, *S. enterica*, and *Pseudomonas* species. However, unlike the other members, MCR‐5 is associated with intact Tn*3*‐type transposition elements that would allow it to mobilize onto plasmids or chromosomes. Here, our study identifies *mcr‐5* on a large nonconjugative plasmid from *A. hydrophila* that is missing the characteristic repeats flanking the transposon but retains the transposase (*tnpA*) and resolvase (*tnpR*) genes. During the progress of this work, another *mcr‐5*‐carrying *A. hydrophila* strain, I064‐2, with a pig origin was reported in China.^38^ Unfortunately, the genome of strain I064‐2 was not available for comparison. However, *mcr‐5* was carried by a small (7915 bp) ColE2‐type plasmid pI064‐2. Unlike the *mcr‐5*‐carrying Tn*3* family transposon on pMCR5_045096 in this study, the *tnpA* and *tnpR* genes and one IRR of the Tn*3* family transposon were missing but the IRL was present on pI064‐2 (Figure 2). This indirectly captures MCR‐5 in different stages of mobilization via the “paste and copy” mechanism of Tn*3* transposition and suggests that pI064‐2 might have been formed later than pMCR5_045096. Genetic analyses suggest that the current diversity in *mcr‐1* originated from an IS*Apl1* based transposition event, with the loss of the insertion sequences leading to a stabilization of *mcr‐1*. The source of diversity in the MCR family and the evolutionary pressures driving them have never been clearly demonstrated.

The Moraxellaceae family has been suggested to be a chromosomal reservoir for the MCR‐1/2 family.^44^, ^47^, ^48^ Similar observations have been made between MCR‐3 and *Aeromonas*. Given that Aeromonads are prevalent in the aquatic ecosystem and that colistin is used extensively in aquaculture, this is quite reasonable. The discovery of MCR‐5 in *Aeromonas* is concerning when combined with the fact that unlike the rest of the MCR family, both MCR‐3 and MCR‐5 impart lower resistance to colistin in an *E. coli* model. The phylogenetic data from this study also seem to indicate that MCR‐5 and MCR‐3/4 might have evolved from a common ancestor that is itself distinct from the MCR‐1/2 family, whose structural domains are incompatible with that of MCR‐5 (Figure 8). More data are, however, necessary to conclusively determine the shared origins of this branch of the MCR family.

However, despite the evolutionary differences, the entire MCR family seems to share significant similarities in the architecture and composition of the active site cavity and a paralleled catalytic mechanism. This cavity is formed at the domain interface and is positioned close to the inner membrane, possibly to access the PE‐lipid substrate. Both the in vivo and in vitro data point toward a “ping‐pong” mode of catalysis, wherein the enzyme reaction proceeds in two steps (Figure S6, Supporting Information), similar to MCR‐1/2/3 does.^10^, ^11^, ^23^, ^44^ The PEA group is cleaved from the first substrate, PE‐lipid, and covalently bound to an active site threonine. This PEA group is then transferred to the suggestive 4′‐phosphate of lipid A to form PPEA‐4′‐lipid A. However, a clear cavity can accommodate the lipid A that is not obvious and has not been elucidated. However, this modified lipid A has been unequivocally demonstrated in vivo for the entire MCR family by subjecting purified lipid A extracts of MCR‐expressing strains to mass spectrometry. Our recent explorations have already indicated that this modification of lipid A alleviates the stress of ROS elicited by colistin exposure.^23^, ^44^ As predicted, similar scenario was seen with MCR‐5 action, in which ROS formation proceeds via Fenton reaction (Figure S8A,B, Supporting Information) and can be specifically bypassed upon the addition of either the ferric chelator bipyridine or a universal ROS scavenger l‐cysteine (Figure S8C, Supporting Information). Apparently, this is distinct from other chromosomal modifications that result in colistin resistance such as addition of cationic sugars to lipid A^49^ or glycine to glucosamine residues.^50^

Taken together, the characterization of MCR‐5 action represents a functional proof for the rapidly evolving family of mobile colistin resistance. The proposal that an entire MCR family is functionally unified, constitutes a significant step toward curtailing the rapid evolution and spread of colistin resistance. In terms of detailed perspectives from genomic, evolutionary, structural, and mechanistic studies, we are allowed to be brought closer in the context of developing novel therapeutic agents and adjuvants that can address the whole MCR family.

## 4 Experimental Section

*Isolation and Identification of A. hydrophila*: The strain WCHAH045096 of *A. hydrophila* was isolated from hospital sewage, which was collected from the influx of the wastewater treatment plant at West China Hospital, Sichuan University, Chengdu, China, in October 2014. Species identification was performed using partial sequencing of the *gyrB* gene as described previously.^51^

*Strains, Plasmids, and Growth Conditions*: Except for *A. hydrophila*, all the other strains referred to the derivatives of *E. coli* MG1655 (Table S1, Supporting Information). Primers were designed for gene cloning and/or PCR detection (Table S1, Supporting Information). The *mcr‐5* gene was amplified with PCR from *A. hydrophila*, and then cloned into two expression vectors (an arabinose‐inducible vector pBAD24 and the IPTG‐inducible plasmid pET21a), giving pBAD24.8xHis*::mcr‐5*, and pET21*::mcr‐5*, respectively. As we recently described^10^, ^11^ with little change, all the point mutants of *mcr‐5* were generated using site‐directed mutagenesis with the Mut Express II fast mutagenesis kit V2 (Vazyme Biotech Co., Ltd.). Overlapping PCR experiments were performed to create the domain‐swapped versions of *mcr‐5/2/1*. Besides the two hybrid versions (TM1‐MCR‐2 and TM2‐MCR‐1) we developed earlier,^21^ four more mosaic genes involved TM1‐MCR‐5, TM5‐MCR‐1, TM2‐MCR‐5, and TM5‐MCR‐2. As a result, all the plasmid constructs were confirmed with direct DNA sequencing as well as PCR detections. The engineered *E. coli* strains were cultivated at 37 °C, in which either liquid Luria‐Bertani (LB) broths or LB agar plates are involved.^9^, ^10^

*Bacterial Conjugation*: Conjugation experiments were carried out both in broth and on filters. The azide‐resistant *E. coli* strain J53 and a colistin‐susceptible azide‐resistant *A. hydrophila* strain both were used as the recipient. Possible trans‐conjugants were selected on LB agar plates containing 2 µg mL^−1^ colistin and 150 µg mL^−1^ azide.

*Determination of Colistin Susceptibility*: MIC of colistin was determined using the microdilution method following the recommendations of the Clinical Laboratory Standards Institute (CLSI).^52^ As there are no breakpoints of colistin from CLSI, those defined by European Committee on Antimicrobial Susceptibility Testing (EUCAST, http://www.eucast.org/) were applied. The strains tested here included *A. hydrophila* and the engineered versions of *E*. *coli* carrying *mcr*‐like genes (Table S1, Supporting Information). No less than three independent trials were conducted as recently described.^46^

*Whole Genome Sequencing*: Genomic DNA of strain WCHAH045096 was prepared using the QIAamp DNA mini kit (Qiagen, Hilden, Germany) and was subjected to whole genomic sequencing using both Illumina HiSeq 2500 platform (Illumina, San Diego, CA) and the long‐read MinION Sequencer (Nanopore, Oxford, UK). The de novo hybrid assembly of both short Illumina reads and long MinION reads was performed using Unicycler v0.4.3^53^ under conservative mode for an increased accuracy. Complete circular contigs generated were then corrected using Pilon v1.22^54^ with Illumina reads for several rounds until no change was detected. Antimicrobial resistance genes were identified from genome sequences using the ABRicate program (https://github.com/tseemann/abricate) to query the ResFinder database (https://cge.cbs.dtu.dk/services/ResFinder/). Plasmid replicon type and plasmid multilocus sequence type were determined using the PlasmidFinder (https://cge.cbs.dtu.dk/services/PlasmidFinder/) and pMLST tools (https://cge.cbs.dtu.dk/services/pMLST/). The chromosome of *A. hydrophila* WCHAH045096 and the complete sequence of pMCR5_045096 were separately deposited into GenBank under the Accession Nos. CP028568 and CP028567, respectively.

*Preparation and Identification of MCR‐5*: The recombinant version of MCR‐5 integral membrane protein was overexpressed in the strain of BL21 (Rossetta) with pBAD24.8xHis::*mcr‐5* (Table S1, Supporting Information) as recently described with MCR‐1/2/3^9^, ^10^, ^11^, ^21^, ^23^ and ICR‐Mo.^44^ Following three rounds of passages (i.e., once at 500 psi and twice at 1300 psi) through a French press (JN‐Mini, Guangzhou, China), bacterial lysates were subjected to 1 h of ultracentrifugation (38 000 rpm at 4 °C) after 1 h of routine spinning (16 800 rpm at 4 °C). The resultant fraction of precipitates containing the interest MCR‐5 protein was solubilized with Buffer B [20 × 10^−3^ m Tris‐HCl (pH 8.0), 50 × 10^−3^ m NaCl, 5 × 10^−3^ m imidazole, 5% glycerol and 1% detergent DDM (m/v)] and then incubated with pre‐equilibrated Ni‐NTA agarose beads for 4 h at 4 °C for further affinity chromatography. The purified MCR‐5 protein was concentrated with a ultrafilter column (30 kDa cutoff, Millipore), judged by sodium dodecyl sulfate polyacrylamide gel electrophoresis (SDS‐PAGE) (12%), and identified with A Waters Q‐Tof API‐US Quad‐ToF mass spectrometer.^55^, ^56^ The secondary structure of MCR‐5 was elucidated by assaying the spectrum of circular dichroism (CD) recorded on a Jasco model J‐1500 spectrometer (Jasco Corp., Tokyo, Japan). The occupancy of zinc within MCR‐5 was determined using the ICP‐MS measured by a NexION 300TM ICP‐MS instrument (PerkinElmer Life Sciences).^57^

*In Vitro Assays for MCR‐5 Catalysis Reaction*: As initially reported by Anandan et al.^58^ with EptA, an enzymatic reaction system was established to test in vitro activity of MCR‐5. 1‐Acyl‐2‐sn‐glycero‐3‐phosphoethanolamine (NBD‐glycerol‐3‐PEA, Avanti Lipids, USA) acted as an alternative substrate of MCR‐5 enzyme. Similar to that with MCR‐1/2^10^, ^11^ and ICR‐Mo,^44^ was also applied to separate the NBD‐glycerol product from the MCR‐5 reaction mixture and subjected to TLC‐based separation, following ≈20 h of incubation at 25 °C. The product of NBD‐glycerol was distinguished from the substrate of NBD‐glycerol‐3‐PEA in terms of the difference of their migration rates on TLC. In addition to the known substrate, the identity of MCR‐5 reaction product was also verified with the liquid chromatography mass spectrometry (LC/MS, Agilent Technologies 6460 Triple Quad LC/MS).^59^

*Measurement of Cytosolic ROS Level*: Prior to the challenge with colistin (2 µg mL^−1^, 0.5 h), the mid‐log phase cultures (OD600, 0.5) of *E. coli* alone or carrying *mcr‐1/5* variants were stained with the oxidant sensor dye DCFH2‐DA (Sigma) (10 × 10^−3^ m, 0.5 h) to detect the intracellular ROS.^60^ Subsequently, bacterial samples were diluted into 10^6^ CFU and subjected to confocal microscopy (and/or flow cytometry) based measurement of cytosolic ROS.^60^

*Structural Determination of LPS‐Lipid A*: LPS‐lipid A pools were isolated and purified from the engineered *E. coli* with or without *mcr‐5* (or its derivatives) as Liu et al.^12^, ^61^ described with little modification. Of particular note, the crude LPS in 10 × 10^−3^ m sodium acetate buffer (pH 4.5) with aqueous 0.2% SDS was kept at 100 °C for 1 h to cleave the Kdo linkage, giving the purified lipid A species.^62^ The purity of lipid A separated with SDS‐PAGE was checked using sensitive silver staining along with SDS‐PAGE.^63^ The qualified lipid A species were subjected to structural identification with matrix‐assisted laser desorption ionization time of flight (MALDI‐TOF) mass spectrometry (Bruker, ultrafle Xtreme).^50^, ^64^ In general, each of MS spectra here was produced with an average of 500 shots and 50% laser power.

*Structural Modeling and Molecular Docking*: The architecture of MCR‐5 in full length was modeled with Swiss‐Model (https://swissmodel.expasy.org/interactive/qMEvX5/models/),^65^ and the EptA of *Neisseria meningitidis* [PDB: 5FGN]^58^ functioned as structural template. Of note, the value of both coverage and QMEAN (that provides a global and local absolute quality estimate on the modeled structure^66^) allowed to judge whether this is a suitable prediction or not.

The binding of MCR‐5 to its phosphatidylethanolamine (PE) lipid substrate was predicted through molecular docking with UCSF DOCK 6.7 software (version 6.7).^67^ The ready‐to‐dock 3D structure of PE (ID: ZINC32837871) and its head group (ID: ZINC02798545) was derived from ZINC database.^68^ Protein structure was concretely optimized for molecular docking using UCSF Chimera software.^69^ The diagram for 2D ligand–protein interaction was generated using LigPlot+ software.^70^

*Phylogenetic Analyses*: The MCR family of colistin resistance enzymes was downloaded from GenBank database, which covers no less than five different subtypes (i.e., MCR‐1,^9^, ^12^, ^71^ MCR‐2,^21^, ^22^ MCR‐3,^23^, ^24^ MCR‐4,^25^, ^26^, ^27^, ^72^ and MCR‐5^13^, ^25^, ^26^, ^28^). Each subtype involves an array of heterogeneous variants as recently stated.^72^ The evolutionary history of MCR‐5 was inferred using the maximum likelihood method. The trees presented here were inferred from 1000 bootstrap replicates using a LG amino acid substitution model. The percentages of replicate trees in which the associated taxa are clustered in the bootstrap test (1000 replicates) are shown next to the branches. A discrete gamma distribution was used to model evolutionary rate differences among sites with some evolutionarily invariable sites. Protein accession numbers of individual members are indicated in the figure. The well‐studied intrinsic EptA from *N. meningitidis* 58 and the inactive Z1140 of *E. coli* O157:H7 EDL933^23^ are included as internal references.

## Conflict of Interest

The authors declare no conflict of interest.

## Supporting information

Supplementary
