# Supplementary material for: A Genomic, Evolutionary, and Mechanistic Study of MCR‐5 Action Suggests Functional Unification across the MCR Family of Colistin Resistance
Source: Adv Sci (Weinh). 2019 Apr 3;6(11):1900034. doi: 10.1002/advs.201900034 (PMC6548960; doi:10.1002/advs.201900034)
Supplement: Supplementary file 1 — Supplementary [file ADVS-6-1900034-s001.pdf]

Copyright WILEY-VCH Verlag GmbH & Co. KGaA, 69469 Weinheim, Germany, 2019.

ADVANCED SCIENCE
Open Access

Supporting Information

for *Adv. Sci.*, DOI: 10.1002/advs.201900034

A Genomic, Evolutionary, and Mechanistic Study of MCR-5 Action Suggests Functional Unification across the MCR Family of Colistin Resistance

Huimin Zhang, Zhiyong Zong, Sheng Lei, Swaminath Srinivas, Jian Sun, Yu Feng, Man Huang, and Youjun Feng**

Supplementary Information

Table S1 Bacterial strains, plasmids and primers used in this study

Strain or plasmids	Relevant characteristics	Origins
Strains		
DH5α	A cloning host of *E. coli*	Lab stock
MG1655	A wild-type strain of *E. coli*	Lab stock
FYJ795	MG1655 carrying pBAD24::*mcr-1*	Lab stock
FYJ855	MG1655 carrying pBAD24::*mcr-2*	Lab stock
FYJ1125	MG1655 carrying pBAD24::*mcr-3*	Lab stock
FYJ1049	MG1655 carrying pBAD24::*mcr-4*	Lab stock
FYJ1303	MG1655 carrying pBAD24::*mcr-5*	This work
FYJ1304	MG1655 carrying pBAD24::*mcr-5* (N112A)	This work
FYJ1305	MG1655 carrying pBAD24::*mcr-5* (T116A)	This work
FYJ1306	MG1655 carrying pBAD24::*mcr-5* (E120A)	This work
FYJ1307	MG1655 carrying pBAD24::*mcr-5* (E248A)	This work
FYJ1308	MG1655 carrying pBAD24::*mcr-5* (T286A)	This work
FYJ1309	MG1655 carrying pBAD24::*mcr-5* (S331A)	This work
FYJ1310	MG1655 carrying pBAD24::*mcr-5* (K334A)	This work
FYJ1311	MG1655 carrying pBAD24::*mcr-5* (H384A)	This work
FYJ1312	MG1655 carrying pBAD24::*mcr-5* (H389A)	This work
FYJ1313	MG1655 carrying pBAD24::*mcr-5* (D458A)	This work
FYJ1314	MG1655 carrying pBAD24::*mcr-5* (H459A)	This work
FYJ1315	MG1655 carrying pBAD24::*mcr-5* (H471A)	This work
FYJ1316	MG1655 carrying pBAD24::tm(*mcr-1*)-*mcr-5*	This work
FYJ1317	MG1655 carrying pBAD24::tm(*mcr-5*)-*mcr-1*	This work
FYJ1318	MG1655 carrying pBAD24::tm(*mcr-2*)-*mcr-5*	This work
FYJ1319	MG1655 carrying pBAD24::tm(*mcr-5*)-*mcr-2*	This work
FYJ1151	BL21 carrying pET21a::*mcr-1*	Lab stock
FYJ916	BL21(pLysS) carrying pET21a::*mcr-2*	Lab stock
FYJ1320	BL21(pLysS) carrying pBAD24.8xHis::*mcr-5*	This work
FYJ917	BL21(pLysS) carrying pET21a::tm(*mcr-1*)-*mcr-2*	This work
FYJ918	BL21(pLysS) carrying pET21a::tm(*mcr-2*)-*mcr-1*	This work
FYJ1320	BL21(pLysS) carrying pBAD24.8xHis::tm(*mcr-1*)-*mcr-5*	This work
FYJ1321	BL21(pLysS) carrying pBAD24.8xHis::tm(*mcr-5*)-*mcr-1*	This work
FYJ1322	BL21(pLysS) carrying pBAD24.8xHis::tm(*mcr-2*)-*mcr-5*	This work
FYJ1323	BL21(pLysS) carrying pBAD24.8xHis::tm(*mcr-5*)-*mcr-2*	This work

FYJ1324	BL21(pLysS) carrying pBAD24.8xHis*::mcr-5* (N112A)	This work
FYJ1325	BL21(pLysS) carrying pBAD24.8xHis*::mcr-5* (T116A)	This work
FYJ1326	BL21(pLysS) carrying pBAD24.8xHis*::mcr-5* (E120A)	This work
FYJ1327	BL21(pLysS) carrying pBAD24.8xHis*::mcr-5* (E248A)	This work
FYJ1328	BL21(pLysS) carrying pBAD24.8xHis*::mcr-5* (T286A)	This work
FYJ1329	BL21(pLysS) carrying pBAD24.8xHis*::mcr-5* (S331A)	This work
FYJ1330	BL21(pLysS) carrying pBAD24.8xHis*::mcr-5* (K334A)	This work
FYJ1331	BL21(pLysS) carrying pBAD24.8xHis*::mcr-5* (H384A)	This work
FYJ1332	BL21(pLysS) carrying pBAD24.8xHis*::mcr-5* (H389A)	This work
FYJ1333	BL21(pLysS) carrying pBAD24.8xHis His*::mcr-5* (D458A)	This work
FYJ1334	BL21(pLysS) carrying pBAD24.8xHis*::mcr-5* (H459A)	This work
FYJ1335	BL21(pLysS) carrying pBAD24.8xHis*::mcr-5* (H471A)	This work
**Plasmids**		
pBAD24	Arabinose inducible promoter-driven expression vector; Amp^R	Lab stock
pBAD24*::mcr-5*	A pBAD24 carrying the wild-type of *mcr-1* at the two cuts of EcoRI and SalI; Amp^R	This work
pBAD24*::mcr-5*(N112A)	pBAD24 encoding the mutant of *mcr-5* (N112A); Amp^R	This work
pBAD24*::mcr-5*(T116A)	pBAD24 encoding the mutant of *mcr-5* (T116A); Amp^R	This work
pBAD24*::mcr-5*(E120A)	pBAD24 encoding the mutant of *mcr-5* (E120A); Amp^R	This work
pBAD24*::mcr-5*(E248A)	pBAD24 encoding the mutant of *mcr-5* (E248A); Amp^R	This work
pBAD24*::mcr-5*(T286A)	pBAD24 encoding the mutant of *mcr-5* (T286A); Amp^R	This work
pBAD24*::mcr-5*(S331A)	pBAD24 encoding the mutant of *mcr-5* (S331A); Amp^R	This work
pBAD24*::mcr-5*(K334A)	pBAD24 encoding the mutant of *mcr-5* (K334A); Amp^R	This work

pBAD24::*mcr-5*(H384A)	pBAD24 encoding the mutant of *mcr-5* (H384A); Amp^R	This work
pBAD24::*mcr-5*(H389A)	pBAD24 encoding the mutant of *mcr-5* (H389A); Amp^R	This work
pBAD24::*mcr-5*(D458A)	pBAD24 encoding the mutant of *mcr-5*(D458A); Amp^R	This work
pBAD24::*mcr-5*(H459A)	pBAD24 encoding the mutant of *mcr-5*(H459A); Amp^R	This work
pBAD24::*mcr-5*(H471A)	pBAD24 encoding the mutant of *mcr-5*(H471A); Amp^R	This work
pBAD24::tm(*mcr-1*)-*mcr-5*	pBAD24 encoding the chimeric version of *mcr-5* whose transmembrane region is replaced with counterpart of *mcr-1*; Amp^R	This work
pBAD24::tm(*mcr-5*)-*mcr-1*	pBAD24 encoding the chimeric version of *mcr-1* whose transmembrane region is replaced with counterpart of *mcr-5*; Amp^R	This work
pBAD24::tm(*mcr-2*)-*mcr-5*	pBAD24 encoding the chimeric version of *mcr-5* whose transmembrane region is replaced with counterpart of *mcr-2*; Amp^R	This work
pBAD24::tm(*mcr-5*)-*mcr-2*	pBAD24 encoding the chimeric version of *mcr-2* whose transmembrane region is replaced with counterpart of *mcr-5*; Amp^R	This work
pBAD24.8xHis	Arabinose inducible promoter-driven expression vector; C-terminal 8xHis tag, Amp^R	Lab stock
pBAD24.8xHis::*mcr-5*	A pBAD24.8xHis carrying the wild-type of *mcr-5* at the two cuts of EcoRI and SalI; Amp^R	This work
pBAD24.8xHis::*mcr-5* (N112A)	A pBAD24.8xHis carrying the mutant of *mcr-5* (N112A) at the two cuts of EcoRI and SalI; Amp^R	This work
pBAD24.8xHis::*mcr-5* (T116A)	A pBAD24.8xHis carrying the mutant of *mcr-5* (T116A) at the two cuts of EcoRI and SalI; Amp^R	This work
pBAD24.8xHis::*mcr-5* (E120A)	A pBAD24.8xHis carrying the mutant of *mcr-5* (E120A) at the two cuts of EcoRI and SalI; Amp^R	This work
pBAD24.8xHis::*mcr-5* (E248A)	A pBAD24.8xHis carrying the mutant of *mcr-5* (E248A) at the two cuts of EcoRI and SalI; Amp^R	This work
pBAD24.8xHis::*mcr-5* (T286A)	A pBAD24.8xHis carrying the mutant of *mcr-5* (T286A) at the two cuts of EcoRI and SalI; Amp^R	This work

pBAD24.8xHis::*mcr-5* (S331A)	A pBAD24.8xHis carrying the mutant of *mcr-5* (S331A) at the two cuts of EcoRI and SalI; Amp^R	This work
pBAD24.8xHis::*mcr-5* (K334A)	A pBAD24.8xHis carrying the mutant of *mcr-5* (K334A) at the two cuts of EcoRI and SalI; Amp^R	This work
pBAD24.8xHis::*mcr-5* (H384A)	A pBAD24.8xHis carrying the mutant of *mcr-5* (H384A) at the two cuts of EcoRI and SalI; Amp^R	This work
pBAD24.8xHis::*mcr-5* (H389A)	A pBAD24.8xHis carrying the mutant of *mcr-5* (H389A) at the two cuts of EcoRI and SalI; Amp^R	This work
pBAD24.8xHis::*mcr-5* (D458A)	A pBAD24.8xHis carrying the mutant of *mcr-5* (D458A) at the two cuts of EcoRI and SalI; Amp^R	This work
pBAD24.8xHis::*mcr-5* (H459A)	A pBAD24.8xHis carrying the mutant of *mcr-5* (H459A) at the two cuts of EcoRI and SalI; Amp^R	This work
pBAD24.8xHis::*mcr-5* (H471A)	A pBAD24.8xHis carrying the mutant of *mcr-5* (H471A) at the two cuts of EcoRI and SalI; Amp^R	This work
pBAD24.8xHis::tm(*mcr-1*)-*mcr-5*	A pBAD24.8xHis carrying the chimeric version of *mcr-5* [whose transmembrane region is replaced with counterpart of *mcr-1*] at the two cuts of EcoRI and SalI; Amp^R	This work
pBAD24.8xHis::tm(*mcr-5*)-*mcr-1*	A pBAD24.8xHis carrying the chimeric version of *mcr-1* [whose transmembrane region is replaced with counterpart of *mcr-5*] at the two cuts of EcoRI and SalI; Amp^R	This work
pBAD24.8xHis::tm(*mcr-2*)-*mcr-5*	A pBAD24.8xHis carrying the chimeric version of *mcr-5* [whose transmembrane region is replaced with counterpart of *mcr-2*] at the two cuts of EcoRI and SalI; Amp^R	This work
pBAD24.8xHis::tm(*mcr-5*)-*mcr-2*	A pBAD24.8xHis carrying the chimeric version of *mcr-2* [whose transmembrane region is replaced with counterpart of *mcr-5*] at the two cuts of EcoRI and SalI; Amp^R	This work
**Primers**	**Primer sequences**	
pBAD24-*mcr-5*-F (EcoRI)	5'- CCG GAA TTC ATG CGG TTG TCT GCA TTT AT-3'	
pBAD24-*mcr-5*-R (SalI)	5'- ACGC GTC GAC TCA TTG TGG TTG TCC TTT TC-3'	
pBAD24.8His-*mcr-4*-F(	5'- CCG GAA TTC ATG CGG TTG TCT GCA TTT AT-3'	

EcoRI)	
pBAD24.8His-*mcr-4*-R(SalI)	5'- ACGC <u>GTC GAC</u> TTG TGG TTG TCC TTT TC-3
mcr-1-TM-R	5'-AGC GTG GGT ATC AGC ACA TCA TGA CTG CTG AAC GCC ACC A-3'
mcr-1-OS-F	5'-TGG GTC TGT GGC CAG TCA TGT ATG CCA GTT TCT TTC GCG T-3'
mcr-2-TM-R	5'-AGC GTG GGT ATC AGC ACA TCC TGA CTG CTA AAT AGT CCA A-3'
mcr-2-OS-F	5'-TGG GTC TGT GGC CAG TCA TGT ATG CGA GTT TCT TTC GGG T-3'
mcr-5-TM-R (*mcr-1*)	5'-ACG CGA AAG AAA CTG GCA TAC ATG ACT GGC CAC AGA CCC A-3'
mcr-5-OS-F (*mcr-1*)	5'-TGG TGG CGT TCA GCA GTC ATG ATG TGC TGA TAC CCA CGC T-3'
mcr-5-TM-R (*mcr-2*)	5'-ACC CGA AAG AAA CTC GCA TAC ATG ACT GGC CAC AGA CCC A-3'
mcr-5-OS-F (*mcr-2*)	5'-TTG GA CTA TTT AGC AGT CAG GAT GTG CTG ATA CCC ACG CT-3'
mcr-5-N112A-F	5'-ATG CTG CGG GCA CTG ATG GAG ACG GAC GTC AGG-3'
mcr-5-N112A-R	5'-ATC AGT GCC CGC AGC ATG GCC TTG TCG AGA TA-3'
mcr-5-T116A-F	5'-ATC TGA TGG AGG CAG ACG TCA GGG AAG CCA GTG A-3'
mcr-5-T116A-R	5'-GTC TGC CTC CAT CAG ATT CCG CAG CAT GGC CT-3'
mcr-5-E120A-F	5'-GCA GCC AGT GAG CTG TTG CAA TGG AGA ATG CT-3'
mcr-5-E120A-R	5'-AAC AGC TCA CTG GCT GCC CTG ACG TCC GTC TCC A- 3'
mcr-5-E248A-F	5'-ACT GGT TGT CGG GGC AAC CGT CAG GGC GGC TAA T-3'
mcr-5-E248A-R	5'-TTG CCC CGA CAA CCA GTA CGA GAG CAC GAG GA-3'
mcr-5-T286A-F	5'-GAC GGA TGC AGC TAC ATC CCT TCC CTG CAT GT-3'
mcr-5-T286A-R	5'-ATG TAG CTG CAT CCG TCC CGC AAC TGG TGA CA-3'
mcr-5-S331A-F	5'-TAA CCA GGC AGG CTG TAA AGG CGT CTG TGA TG-3'
mcr-5-S331A-R	5'-TAC AGC CTG CCT GGT TAT CGC GCC AGA GAA TG-3'
mcr-5-K334A-F	5'-TGT GCA GGC GTC TGT GAT GGA CTG CCC TTT GA-3'
mcr-5-K334A-R	5'-TCA CAG ACG CCT GCA CAG CCC GAC TGG TTA TCG C-3'
mcr-5-H384A-F	5'-TCG TTC TGG CAA TGC TGG GCA ATC ACG GCC CA-3'
mcr-5-H384A-R	5'-CAG CAT TGC CAG AAC GAT CAG CAT ATC GCT GC-3'

mcr-5-H389A-F	5’-TAT GCT GGG CAA TGC AGG CCC AGC GTA TTT CCA GC-3’
mcr-5-H389A-R	5’-CTG CAT TGC CCA GCA TAT GCA GAA CGA TCA GC-3’
mcr-5-D458A-F	5’-TAC GTT TCC GCA CAT GGG GAA TCG CTC GGC GA-3’
mcr-5-D458A-R	5’-CCA TGT GCG GAA ACG TAC AGC AGC GCC GTG TC-3’
mcr-5-H459A-F	5’-TTC CGA TGC AGG GGA ATC GCT CGG CGA GAA AG-3’
mcr-5-H459A-R	5’-ATT CCC CTG CAT CGG AAA CGT ACA GCA GCG CC-3’
mcr-5-H471A-F	5’-GTA TCT CGC AGG CAT ACC TTA CGT CAT CGC GC-3’
mcr-5-H471A-R	5’-GTA TGC CTG CGA GAT ACA GGC CTT TCT CGC CG-3’

*The underlined letters denote restriction sites.

Supplementary figures

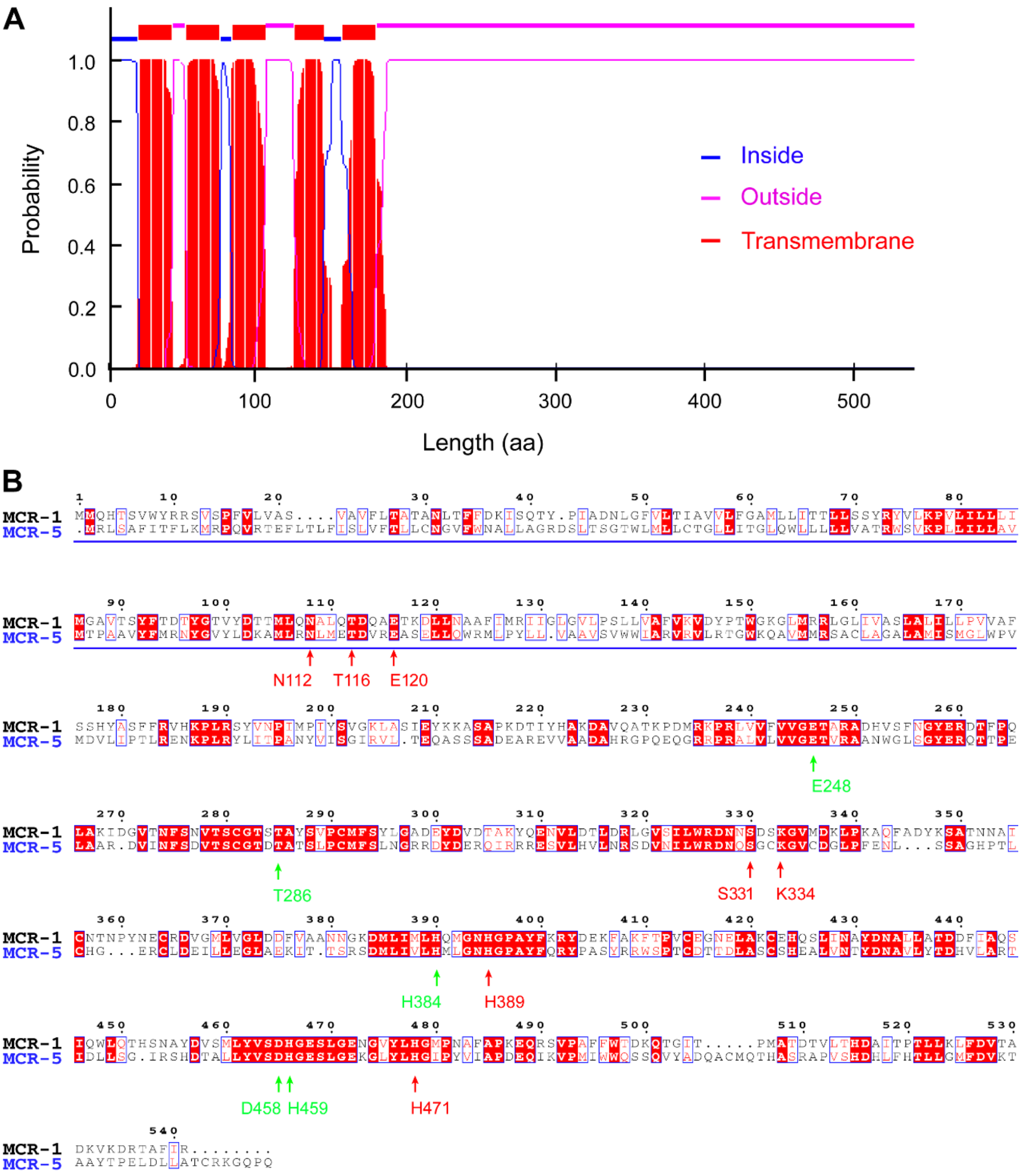

Fig. S1 Bioinformatic analysis of MCR-5

A. Topological prediction of MCR-5 integral membrane protein

B. Sequence alignment of MCR-5 with MCR-1

Transmembrane region prediction is performed with TMHMM Server v. 2.0 (http://www.cbs.dtu.dk/services/TMHMM/). The zinc-interactive residues are indicated in green, and the PE-recognizable residues are colored red.

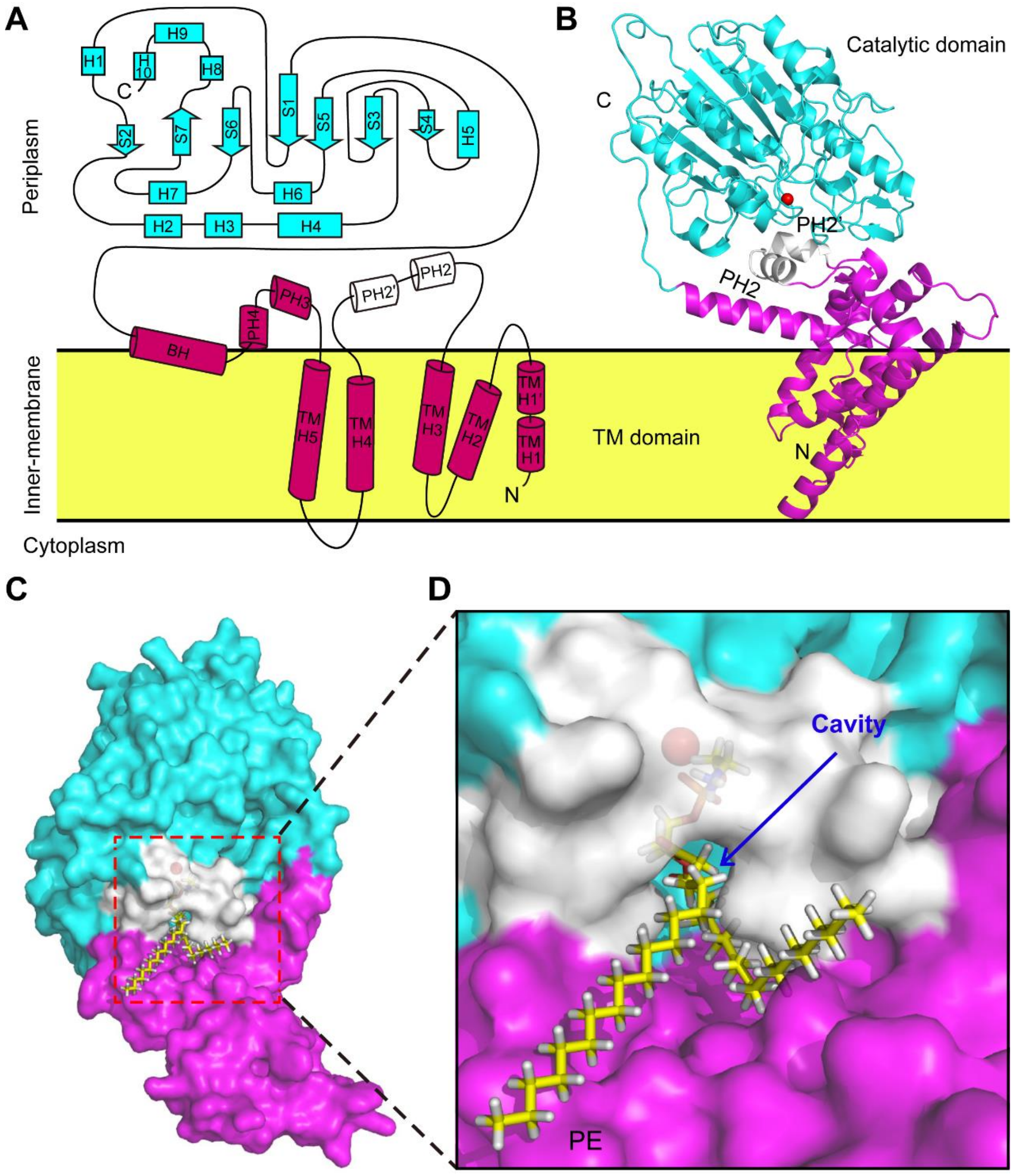

Fig. S2 Structural analyses of MCR-5

A. Topological illustration of MCR-5

B. Ribbon representation for the modelled structure of MCR-5 in full length

C. Surface structure of MCR-5 with a putative PE-recognizable cavity

D. An enlarged view of the putative PE-interactive cavity

The modeled structure is given with PyMol. The TM domain is showed in magenta, the region of catalytic domain appears in blue, and the PE cavity is in white.

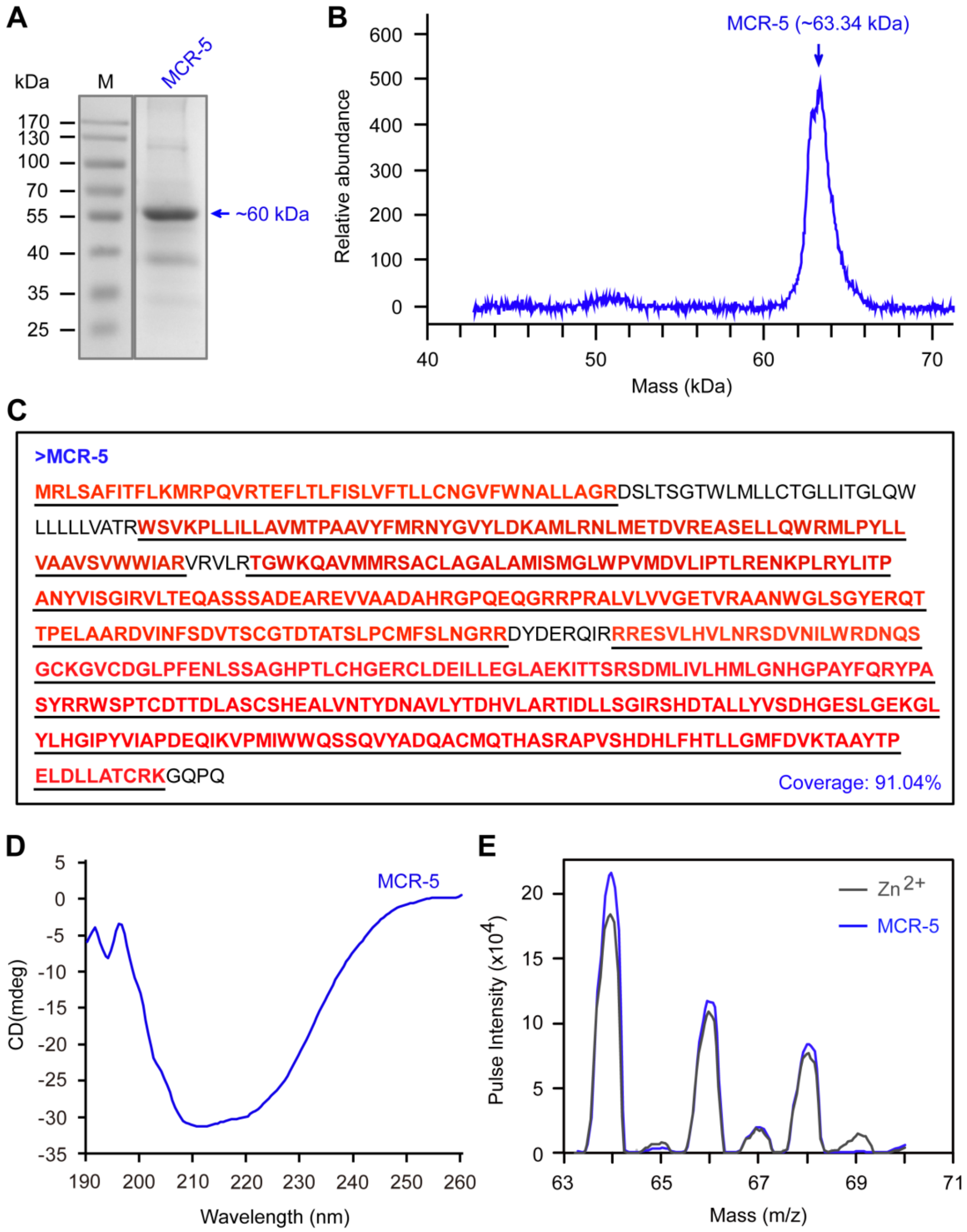

Fig. S3 Biochemical characterization of MCR-5 membrane protein

A. SDS-PGE (12%) profile of the purified MCR-5 protein

B. Use of MS to determine molecular mass of MCR-5 protein

C. MS-based identity of MCR-5

D. Circular dichroism (CD) analyses of MCR-5 indicates a typical spectrum of protein secondary structure rich in α-helix

E. Use of Inductively Coupled Plasma Mass Spectrometry (ICP-MS) to visualize the abundance of zinc occupied with MCR-5

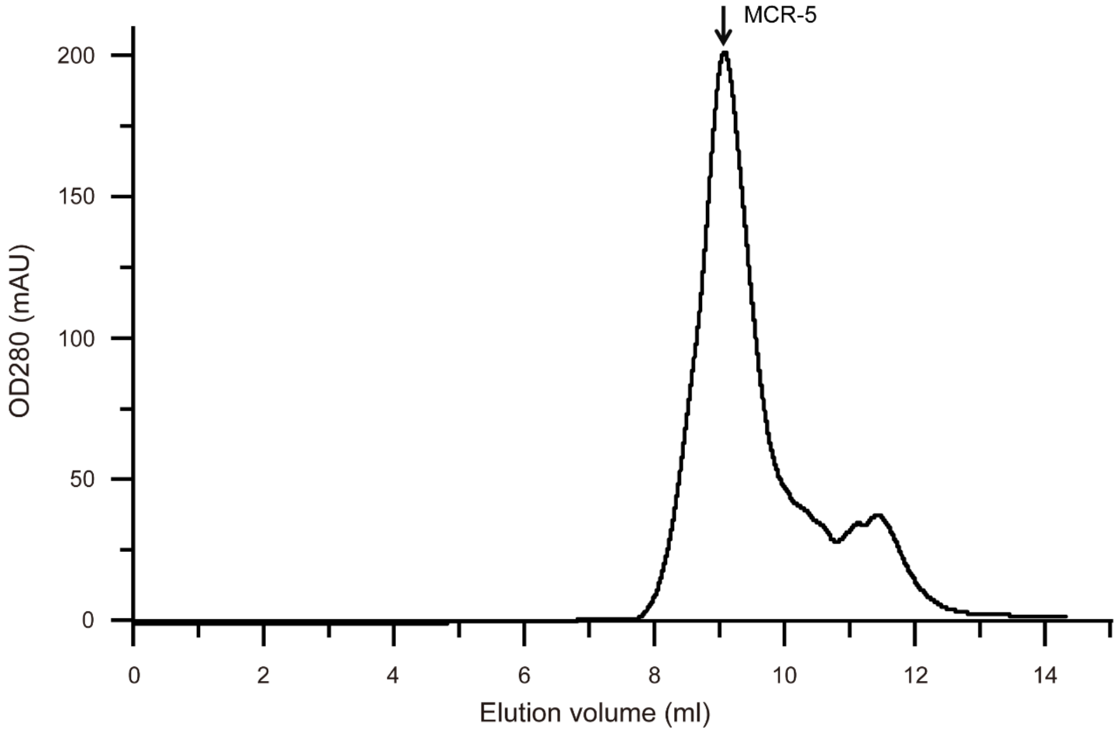

Fig. S4 Gel filtration analyses of MCR-5 protein
Superdex 75 10/300 column is used to separate MCR-5 protein

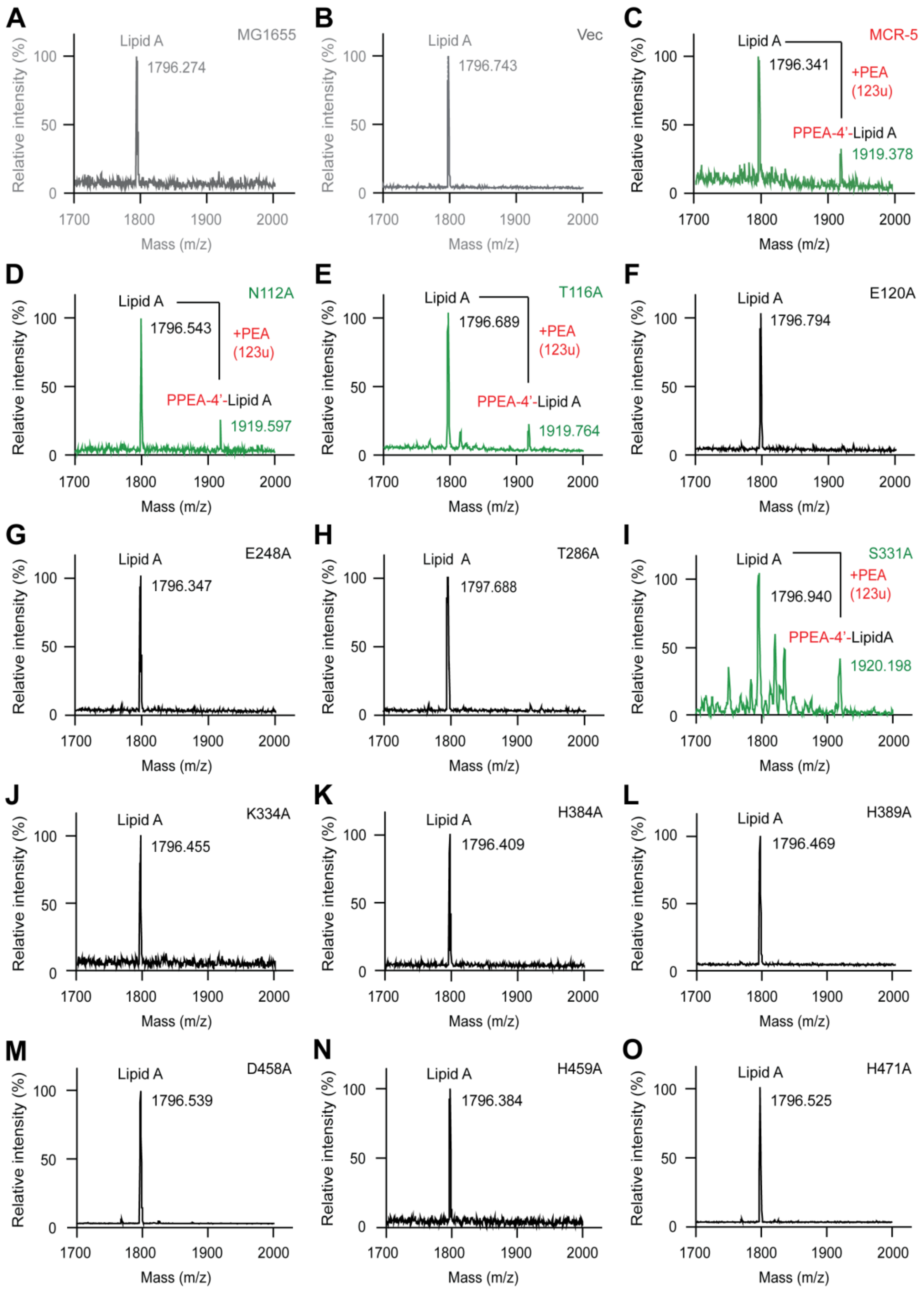

Fig. S5 MALDI-TOF mass spectrometry of lipopolysaccharide (LPS)-lipid A species from *E. coli* expressing *mcr-5* and/or its point-mutants

MS profile of the lipopolysaccharide (LPS)-lipid A species in two negative-controls, the colistin-susceptible strain *E. coli* MG1655 alone (**A**) or

containing the empty vector pBAD24 (**B**)
C. MS spectrum of LPS-lipid A in the positive control strain *E. coli* expressing MCR-5
In addition to the native form of lipid A (m/z, 1796.341), PPEA-4'-lipid A (m/z, 1919.378), the modified form of lipid A, is given upon the presence of *mcr-5*.

In the context of the chemical modification of LPS-lipid A moieties, partial enzymatic activity remains in the 3 point-mutants [N112A (**D**), T116A (**E**) and S331A (**I**)] of MCR-5

The three point-mutants of MCR-5 [namely E120A (**F**), E248A (**G**) and T286A (**H**)] has no detectable activity in the transfer of PEA to suggestive 4'-phosphate position of lipid A species.

Six point-mutants of MCR-5 [namely K334A (**J**), H384A (**K**), H389A (**L**), D458A (**M**), H459A (**N**), and H471A (**O**)] are inactive in the enzymatic activity.

Of note, the MS peak of lipid A species appears at m/z of 1796.274~1796.940. Expression of functional (and/or partial active) versions of *mcr-5* in *E. coli* results in the presence of its modified form PPEA-4'-lipid A at m/z of 1919.378~1920.198. Vec is pBAD24.

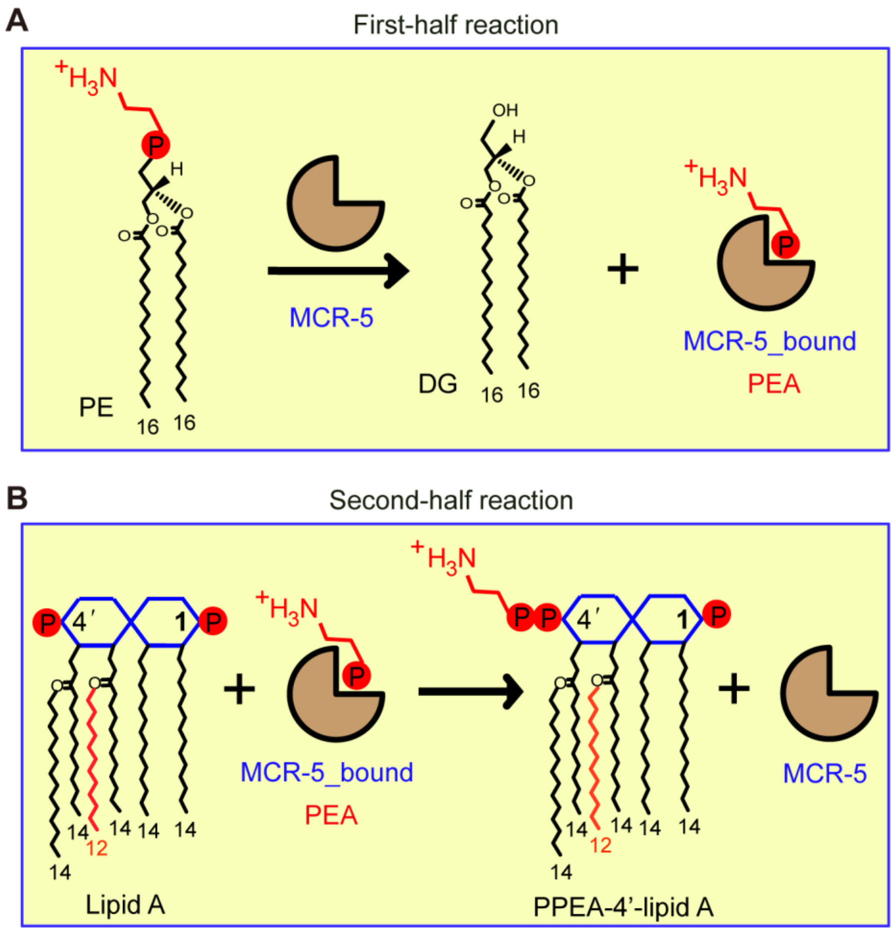

Fig. S6 A working model of "ping-pong" reaction mechanism assigned to MCR-5 action

A. A scheme for the first-half reaction of MCR-5 action in which the PEA moiety is removed from donor substrate PE, giving an intermediate of MCR-5_bound PEA

B. A cartoon representative for the second-half reaction of MCR-5 action, illustrating the transfer of PEA from the adduct of MCR-5_bound PEA to the recipient substrate lipid A and the formation of its resultant PEA-4'-lipid A

It was adapted appropriately from the recently-proposed model for other MCR variants [11, 46, 72].

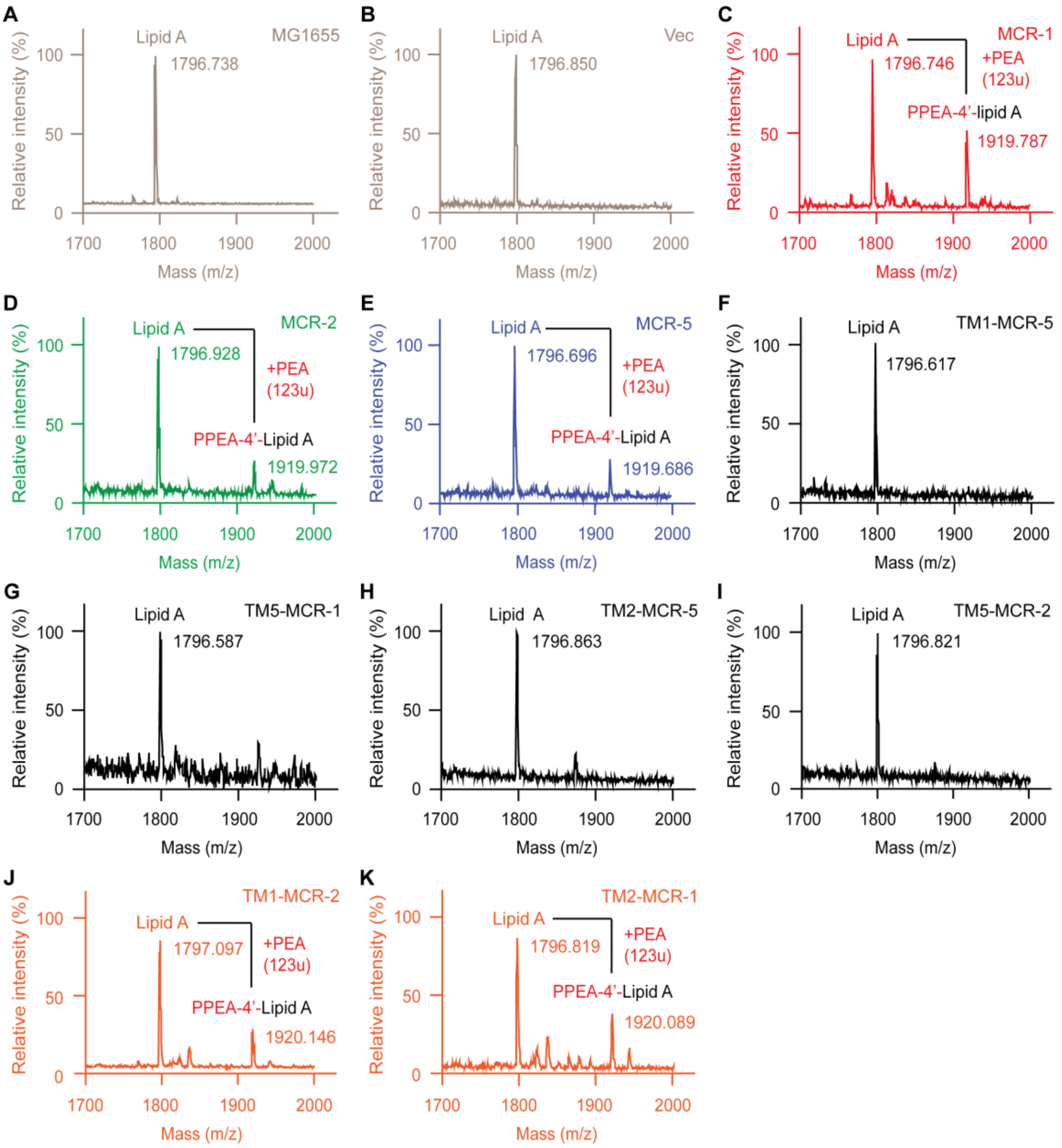

Fig. S7 MS verification for chemical modification of lipid A pools from *E. coli* expressing domain-swapped versions between MCR-5 and MCR-1/2

MS spectrum of the lipopolysaccharide (LPS)-lipid A species in the colistin-susceptible strain *E. coli* MG1655 alone (**A**) or carrying the empty vector pBAD24 (**B**)

MS profiles of LPS-lipid A in the positive control strain *E. coli* harboring MCR-1 (**C**), MCR-2 (**D**), or MCR-5 (**E**)

In addition to the peak of the unmodified lipid A (m/z, 1796.696~1796.928), PPEA-4'-lipid A (m/z, 1919.686~1919.972), a modified form of lipid A, consistently appears upon the presence of *mcr-1*, *mcr-2*, or *mcr-5*.

No enzymatic activity of the chemical modification of LPS-lipid A moieties, is detected in the following four domain-swapped version: TM1-MCR-5 (**F**), TM5-MCR-1 (**G**), TM2-MCR-5 (**H**), and TM5-MCR-2 (**I**).

In the context of addition of PEA to the suggestive 4'-phosphate position of lipid A moieties, the domain-swapped versions [TM1-MCR-2 (**J**) and TM2-MCR-1 (**K**)] of MCR-1/2 are functional.

Designations: TM1-MCR-5, a derivative of MCR-5 with TM1 region of MCR-1 in place of its native TM domain; TM5-MCR-1, a hybrid version of MCR-1 whose TM region is replaced with the counterpart in MCR-5; TM2-MCR-5, a mosaic version of MCR-5 whose TM region is exchanged with that of MCR-2; TM5-MCR-2, a hybrid derivative of MCR-2 whose TM region is replaced with that of MCR-5; TM1-MCR-2, a hybrid derivative of MCR-2 whose TM region is replaced with that of MCR-1; and TM2-MCR-1, a derivative of MCR-1 whose TM region is replaced with that of MCR-2. Vec refers to pBAD24.

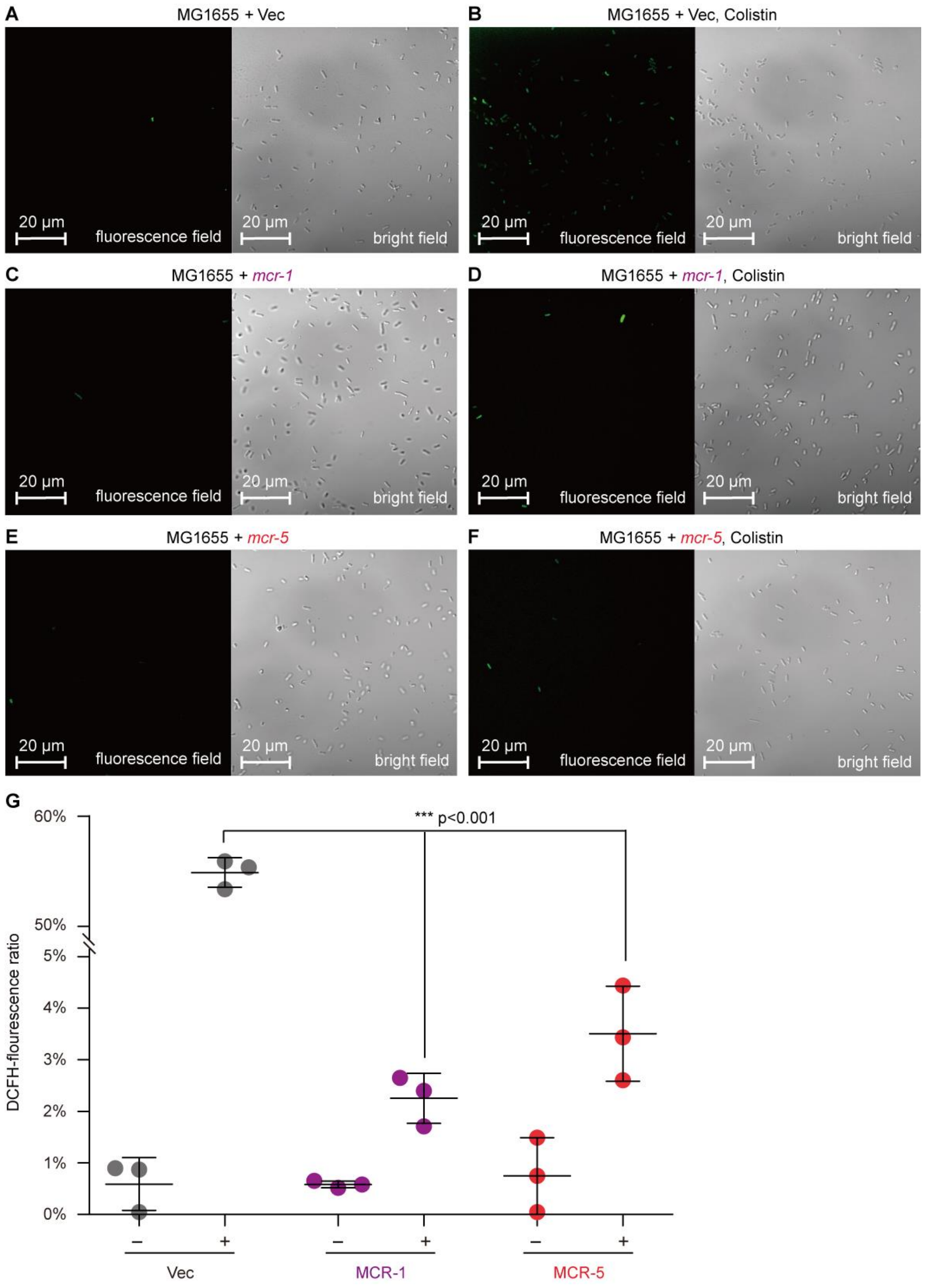

Fig. S8 Use of confocal microscopy to visualize the intracellular ROS accumulated in *E. coli*

A & B The presence of colistin stimulates the formation of hydrogen peroxide in *E. coli* with the empty vector

C & D The expression of MCR-1 interferes colistin-induced production of hydrogen peroxide in *E. coli*

E & F Colistin-triggered production of hydrogen peroxide is attenuated upon the occurrence of MCR-5 in *E. coli*

The intra-cellular ROS level was detected with an oxidant-sensitive dye, DCFH2-DA. The fluorescence was measured with a Zeiss LSM 510 Meta confocal laser scanning microscope (100x oil immersion objective). The hydrogen peroxide produced is denoted in green. The data was expressed using one-way analysis of variance (ANOVA) followed by Tukey-Kramer multiple comparisons post hoc test [47]. Statistical significance was set at *** $p<0.001$.

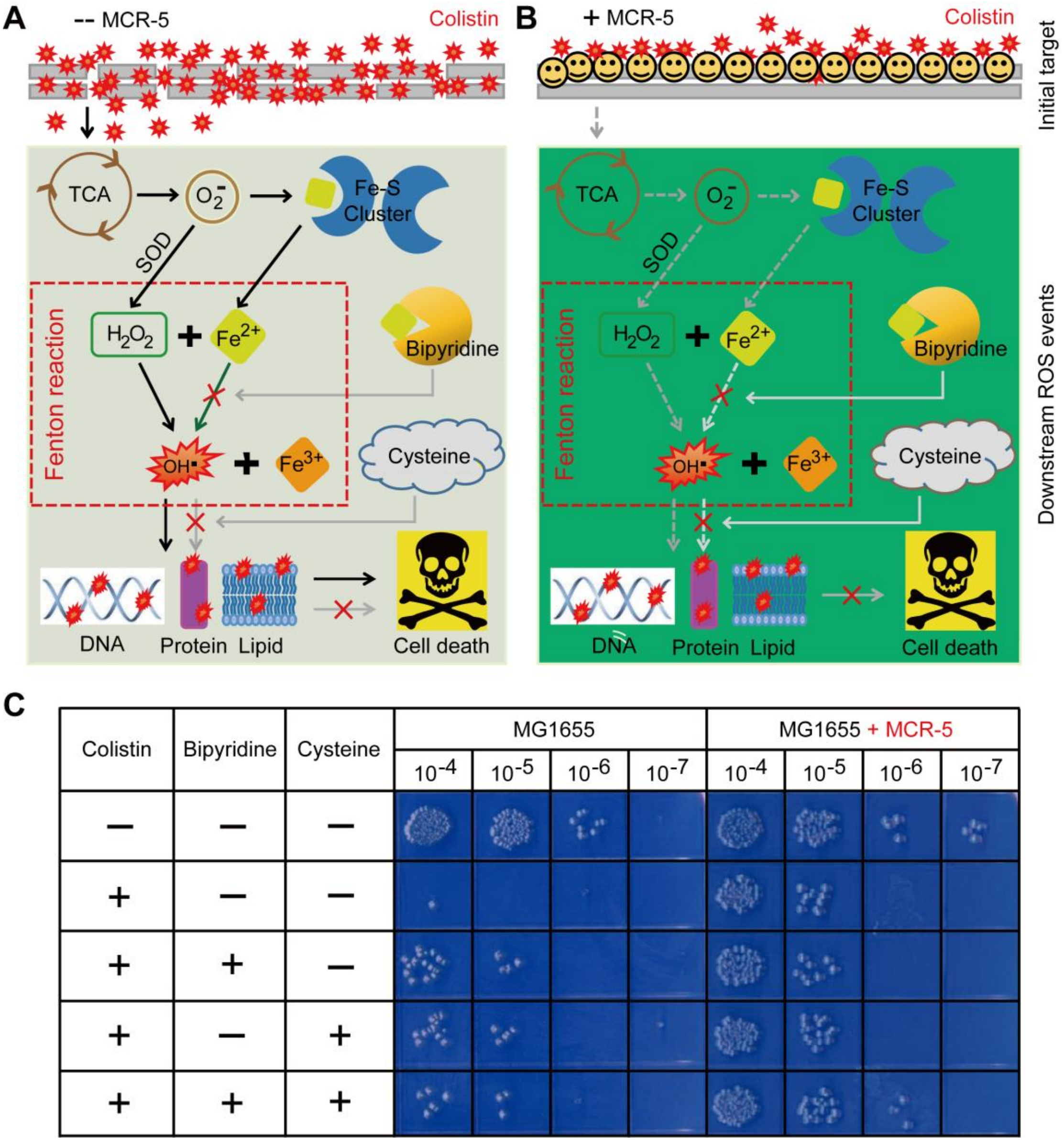

Fig. S9 Functional impairment of the hydroxyl radical death pathway by MCR-5 in *E. coli*

A. A working model for colistin-mediated membrane damage and its downstream ROS formation in *E. coli*

B. A scheme for the presence of MCR-5 rendering the *E. coli* resistant to the entry of colistin, and then to quench the colistin-induced ROS production in *E. coli*

C. Chemical rescue assays validate that Fenton reaction is involved into colistin-triggered hydroxyl radical killing pathway in *E. coli*

A representative result is given from three independent trials.

Of note: The LPS-lipid A denotes an initial target of colistin treatment; bipyridine is a known ferric chelator; and L-cysteine is a common ROS scavenger. The model is adapted from recently proposed by Feng's group [23, 45].

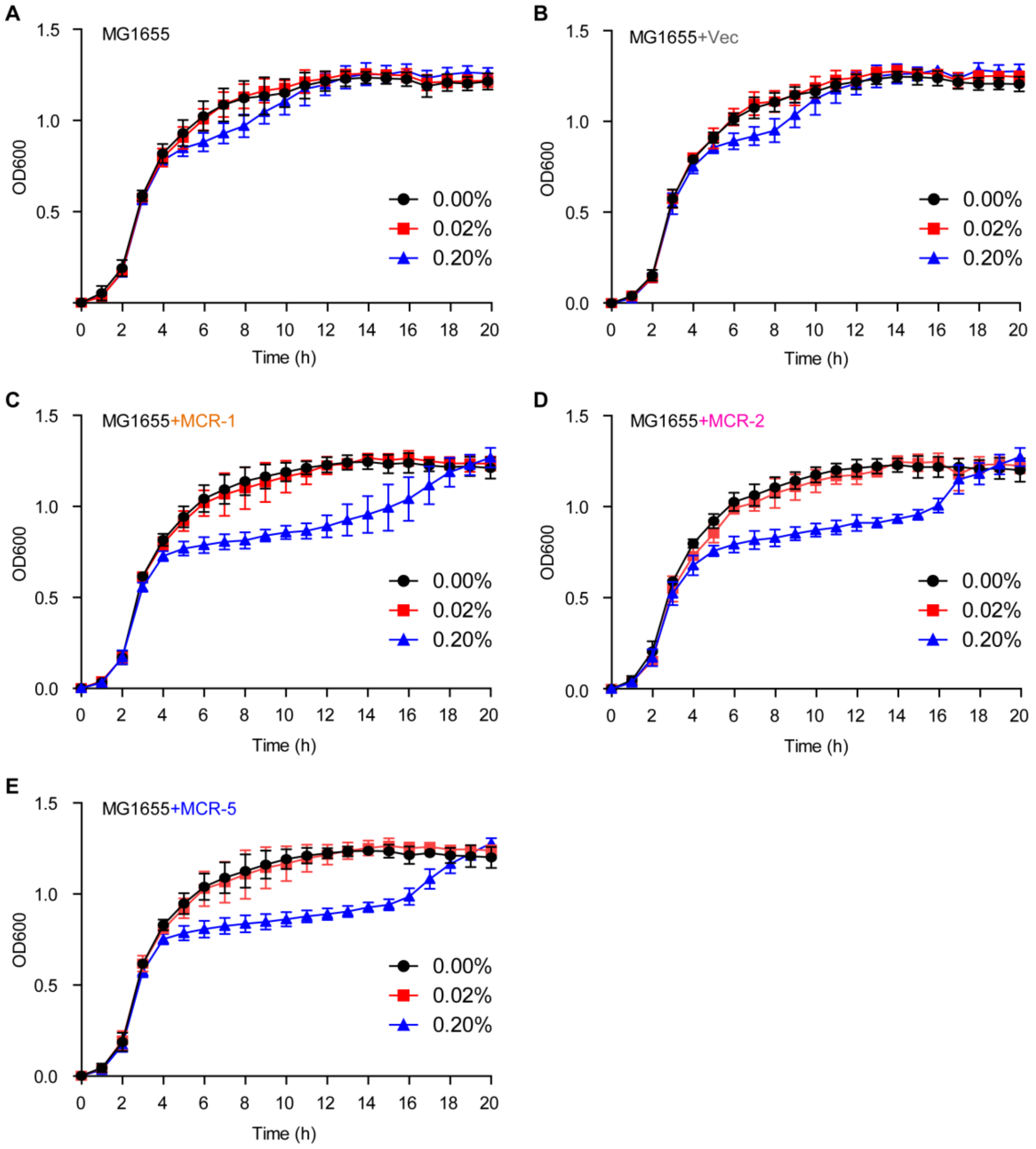

Fig. S10 Interference of bacterial growth by the expression of *mcr-1/2/5*

Growth curves of the two negative control strains [namely the *E. coli* MG1655 alone (**A**) and the empty vector pBAD24-carrying MG1655 (**B**)]
The growth of *E. coli* MG1655 inhibited by the arabinose-induced expression both MCR-1 (**C**) and MCR-2 (**D**)
E. Expression of MCR-5 inhibits on bacterial growth of *E. coli* MG1655
The expression of *mcr-1* (*mcr-2* & *mcr-5*) was triggered through the addition of different levels of arabinose (0.00%, 0.02%, and 0.20%, w/v) into LB media. A representative result is given. Vec denotes pBAD24.
It shows in average ± standard deviations (SD).
